# A synoptic review of the Eocene (Ypresian) cartilaginous fishes (Chondrichthyes: Holocephali, Elasmobranchii) of the Bolca Konservat-Lagerstätte, Italy

**DOI:** 10.1007/s12542-017-0387-z

**Published:** 2017-12-30

**Authors:** Giuseppe Marramà, Giorgio Carnevale, Andrea Engelbrecht, Kerin M. Claeson, Roberto Zorzin, Mariagabriella Fornasiero, Jürgen Kriwet

**Affiliations:** 1 Department of Palaeontology, Geozentrum, University of Vienna, Althanstraβe 14, 1090 Vienna, Austria; 2 Dipartimento di Scienze della Terra, Università degli Studi di Torino, via Valperga Caluso 35, 10125 Turin, Italy; 3 Philadelphia College of Osteopathic Medicine, Philadelphia, PA 19103 USA; 4 Sezione di Geologia e Paleontologia, Museo Civico di Storia Naturale, Lungadige Porta Vittoria 9, 37129 Verona, Italy; 5 Museo di Geologia e Paleontologia, Università di Padova, Via Giotto 1, 35121 Padua, Italy

**Keywords:** Neoselachii, Holocephali, Ypresian, Diversity, Konservat-Lagerstätte, Tethys, Neoselachii, Holocephali, Ypresium, Diversität, Konservat-Lagerstätte, Tethys

## Abstract

Here, we review and discuss the records and taxonomy of the Ypresian (Eocene) chondrichthyans from the famous Bolca Konservat-Lagerstätte in northeastern Italy. Despite the outstanding diversity and the numerous studies focusing on the actinopterygian faunas from Pesciara and Monte Postale, the current knowledge about the systematics, taxonomy and phylogenetic relationships of the cartilaginous fishes from these Eocene sites remains elusive and largely inadequate. The celebrated Eocene Bolca Lagerstätte has yielded several exquisitely preserved articulated remains of chondrichthyan fishes in which delicate structures and soft tissues are preserved, as well as isolated teeth. The cartilaginous fish assemblage of Bolca comprises at least 17 species-level taxa belonging to 10 families in 6 orders, including selachians (Carcharhiniformes, Lamniformes), batoids (Torpediniformes, Myliobatiformes, Rajiformes) and holocephalans (Chimaeriformes). The occurrence of holocephalans represented by an isolated fin-spine of the chimeroid *Ischyodus* in the Bolca assemblage is reported here for the first time and represents the first record of chimeroids in the Eocene of Italy and also southern Europe. The Bolca chondrichthyan assemblage is remarkably different from those of other contemporaneous Boreal or Tethyan deposits, suggesting that its taxonomic composition is largely influenced by the palaeoenvironmental context. However, this synoptic review also highlights the importance of detailed revisions of all chondrichthyan remains from the Bolca Konservat-Lagerstätten.

## Kurzfassung

Obwohl Knochenfische in der berühmten eozänen (Ypresium) Bolca-Konservatlagerstätte in NE-Italien äußerst divers sind und in den vergangenen Jahren Gegenstand zahlreicher Studien waren, ist der derzeitige Kenntnisstand über die Systematik, Taxonomie und Phylogenie der Knorpelfische aus dieser Lagerstätte überraschend gering und ungenau. In dieser Studie geben wir einen Überblick über die Knorpelfische der Bolca-Konservatlagerstätte und diskutieren ihre Verbreitung und ihre Taxonomie. Aus den beiden Fundstellen Pesciara und Monte Postale stammen einige sehr gut erhaltene Exemplare, bei denen auch sehr feine Strukturen und Weichgewebe fossilisiert sind, sowie isolierte Zähne. Die Knorpelfischfauna von Bolca umfasst mindestens 17 Arten, die zu zehn Familien und sechs Ordnungen gehören wie Haie (Carcharhiniformes, Lamniformes), Rochen (Torpediniformes, Myliobatiformes, Rajiformes) und Chimären. Chimären sind erstmals für Bolca durch einen isolierten Flossenstachel von *Ischyodus* belegt, was gleichzeitig der erste Nachweis für Chimären im Eozän von Italien und Süd-Europa ist. Die Knorpelfischassoziation von Bolca unterscheidet sich in ihrer taxonomischen Zusammensetzung auffällig von anderen tethyalen und auch borealen Faunen. Dies lässt vermuten, dass die taxonomische Zusammensetzung im Wesentlichen durch Umweltbedingungen bestimmt ist. Der hier präsentierte synoptische Überblick der Bolca-Knorpelfische zeigt aber auch deutlich, dass detaillierte Revisionen aller Knorpelfischreste der Bolca-Konservatlagerstätte für ein besseres Verständnis dieser wichtigen eozänen Fundstelle dringend nötig sind.

## Introduction

The Paleogene (ca. 66–23 Ma) represents a critical interval in the development of the current global climatic patterns. With a short-lived thermal maximum that occurred at the Paleocene–Eocene boundary (PETM), in turn followed by extensive greenhouse conditions during the Early Eocene Climatic Optimum, a considerable global warming resulted, which lasted until the end of the middle Eocene (ca. 37 Ma). The PETM subsequently was followed by a late Eocene (ca. 49–34 Ma) transition from greenhouse to icehouse conditions (Zachos et al. 2008). The final cooling phase, which took place at the Eocene–Oligocene boundary (ca. 33.7 Ma), was characterised by declining atmospheric CO_2_ content, long-term deep-sea cooling and establishment of large Antarctic ice sheets, resulting in one of the most dramatic climatic shifts in the Cenozoic (see Zachos et al. 2001; Pagani et al. 2005; Lear et al. 2008). These climatic changes, which persisted into the early Oligocene, resulted in major biotic turnovers in marine and terrestrial faunas and floras (Prothero et al. 2003; Pearson et al. 2008).

Although the general patterns of abiotic disruptions during the Paleogene have been documented extensively (see Culver and Rawson 2000) and the fossil record documents profound changes in both marine and terrestrial ecosystems across the PETM (e.g., Gingerich 2006), little is known about the impact of the global changes on overall biodiversity, particularly on organisms occupying the higher trophic levels in marine ecosystems. In particular, the evolutionary and diversity dynamics of cartilaginous fishes (Chondrichthyes) during the Paleogene Climatic Optimum has received little attention, mostly due to the fact that there are few Paleogene faunas that are not from marginal environments (there are many in the North Sea Basin) or very poorly dated (e.g., Morocco). The taxonomy, systematics and evolutionary history of the Paleogene chondrichthyans in general are problematic because of the lack of comprehensive non-dental morphological studies. Studying fossil chondrichthyan fishes is hindered by the nature of their fossil record, which generally is rather rich, although heavily biased towards isolated teeth (see Cappetta 2012). This is due to their predominantly cartilaginous skeleton that has only little chance to fossilize, so that their taxonomy is mostly based on dental characters. Few Paleogene localities, including the marine Bolca Lagerstätte (Italy) and Grube Unterfeld deposit (Germany), and the non-marine Green River Formation (Wyoming), yielded fully or partially complete articulated skeletal remains of cartilaginous fishes (see e.g., Jaekel 1894; de Carvalho et al. 2004; Hovestadt et al. 2010), which are crucial in order to properly understand their evolutionary trajectories and morphological patterns during this part of the Cenozoic history. Moreover, the fossil record of cartilaginous fishes appears also very strongly biased towards taxa with larger teeth, whereas small teeth are not consistently processed in most deposits as results of taphonomic or collecting biases.

The celebrated Eocene (Ypresian, ca. 50 Ma; Papazzoni et al. 2014a) Bolca Konservat-Lagerstätte from northeastern Italy is one of the few fossiliferous sites in which fossils of chondrichthyan fishes are exquisitely preserved. In addition to isolated teeth, fossils include complete and fully articulated skeletal remains, in which delicate mineralised cartilaginous structures and soft tissues are easily preserved and recognizable. Several recent studies extensively contributed knowledge of the outstanding palaeobiodiversity of this deposit, with more than 230 described species-level taxa (see Carnevale et al. 2014), belonging to a variety of teleost lineages (e.g., anguilliforms, atheriniforms, aulopiformes, beryciforms, clupeiforms, lophiiforms, pleuronectiforms, tetraodontiforms and several other percomorph lineages; Blot 1969; Tyler and Santini 2002; Bannikov 2004, 2006, 2008; Carnevale and Pietsch 2009, 2010, 2011, 2012; Marramà and Carnevale 2015a, b, 2016, 2017a, b; Pfaff et al. 2016; Carnevale et al. 2017). However, the diversity of certain groups as well as many aspects of their evolutionary palaeoecology have been totally neglected or underestimated. In particular, most of the selachians and batoids from Bolca have not been studied using modern methodological approaches. Consequently, a revisionary study of the Eocene sharks, skates and rays from the Bolca Lagerstätte is warranted. The goal of this paper is therefore to provide an overview of the chondrichthyan assemblage in the Eocene Bolca Konservat-Lagerstätte, in order to properly define the diversity of sharks, skates and rays from this celebrated locality and their palaeoecological role and biogeographic affinities.

*Institutional abbreviations*: CNHM, Croatian Natural History Museum, Zagreb; MC, Carnegie Museum, Pittsburgh; MCSNV, Museo Civico di Storia Naturale, Verona; MGP-PD, Museo di Geologia e Paleontologia, Università degli Studi di Padova; MNHN, Muséum National d’Histoire Naturelle, Paris; MSNM, Museo Civico di Storia Naturale, Milano; NHMUK, Natural History Museum, London; NHMW, Naturhistorisches Museum, Vienna.

## Historical notes

Since the sixteenth century, the locality of Bolca and its fossils have yielded materials that stimulated philosophical and scientific discussions. The existence of exquisitely preserved “petrified” fishes in the limestones of Bolca was reported for the first time by the famous botanist and physician Pietro Andrea Mattioli in the third edition of the translation of his “Dioscorides De Materia Medicinale” (Mattioli 1550). Later, several prominent naturalists, including Johann Jakob Scheuchzer, Antonio Vallisneri, Ferdinando Marsili, Anton Lazzaro Moro, Scipione Maffei, Déodat de Dolomieu and Giovanni Arduino extensively discussed the fishes from Bolca and their origins during the eighteenth century (Sorbini 1972; Guerra and Zorzin 2014). Towards the end of the eighteenth century, a cogent debate about the origin and significance of these fossils involved three abbots, Domenico Testa, Alberto Fortis and Giovanni Serafino Volta (Gaudant 1997). The latter identified for the first time several sharks and rays from Bolca in the “Ittiolitologia Veronese” (Volta 1796), representing the earliest treatise on palaeoichthyology, in which the author included the description of more than 120 species, including 4 fossils with a keen likeness to extant sharks *Carcharodon carcharias* and *Stegostoma fasciatus* [both as *Squalus* in Volta (1796)], and the batoids *Raja muricata*, which Volta (1796) considered the same as *Pastinachus* “*Raja*” *sephen,* and *Torpedo* “*Raja*” *torpedo*.

In May 1797, about 600 fossil specimens of the prominent collection of fossils from Bolca assembled by Giovambattista Gazola were illegitimately confiscated by the revolutionary armies of Napoleon that occupied Verona, transported to Paris, and deposited in the MNHN (Frigo and Sorbini 1997; Gaudant 2011; Carnevale et al. 2014). Henry Ducrotay de Blainville (1818) used this collection for his account of 18 fossil fishes that appeared in the “Nouveau Dictionnaire d’Histoire Naturelle”, including the description of some batoids as *Narcobatus giganteus* and *Trygonobatus crassicauda.* However, the first critical analysis of this collection was that of the Swiss naturalist Louis Agassiz who reviewed (Agassiz 1835) the identifications presented by Volta (1796), and later described the fossils in great detail in his monumental “Recherches sur les Poissons Fossiles” (Agassiz 1833–1844). Among the others, Agassiz (1833–1844) named new chondrichthyan taxa (e.g., *Galeus cuvieri*, *Torpedo gigantea*, *Narcopterus bolcanus*, *Trygon oblongus*) based on the material from Bolca. After the publication of Agassiz’s (1835, 1833–1844) works, numerous authors have increased what is known about the diversity of the Bolca fish assemblage. With the exception of a few descriptions and revisions of some chondrichthyan taxa by de Zigno (1874a, b, 1876, 1885) and Bassani (1897), to date, only a single comprehensive account of cartilaginous fishes from Bolca has been written (Jaekel 1894). Furthermore, no systematic studies have been carried out on the Bolca cartilaginous fishes since that time, with the exception of recent revisions of selected taxa (e.g., de Carvalho 2010; Fanti et al. 2016; Marramà et al. 2017a, b).

## Geological setting

**Fig. 1:**
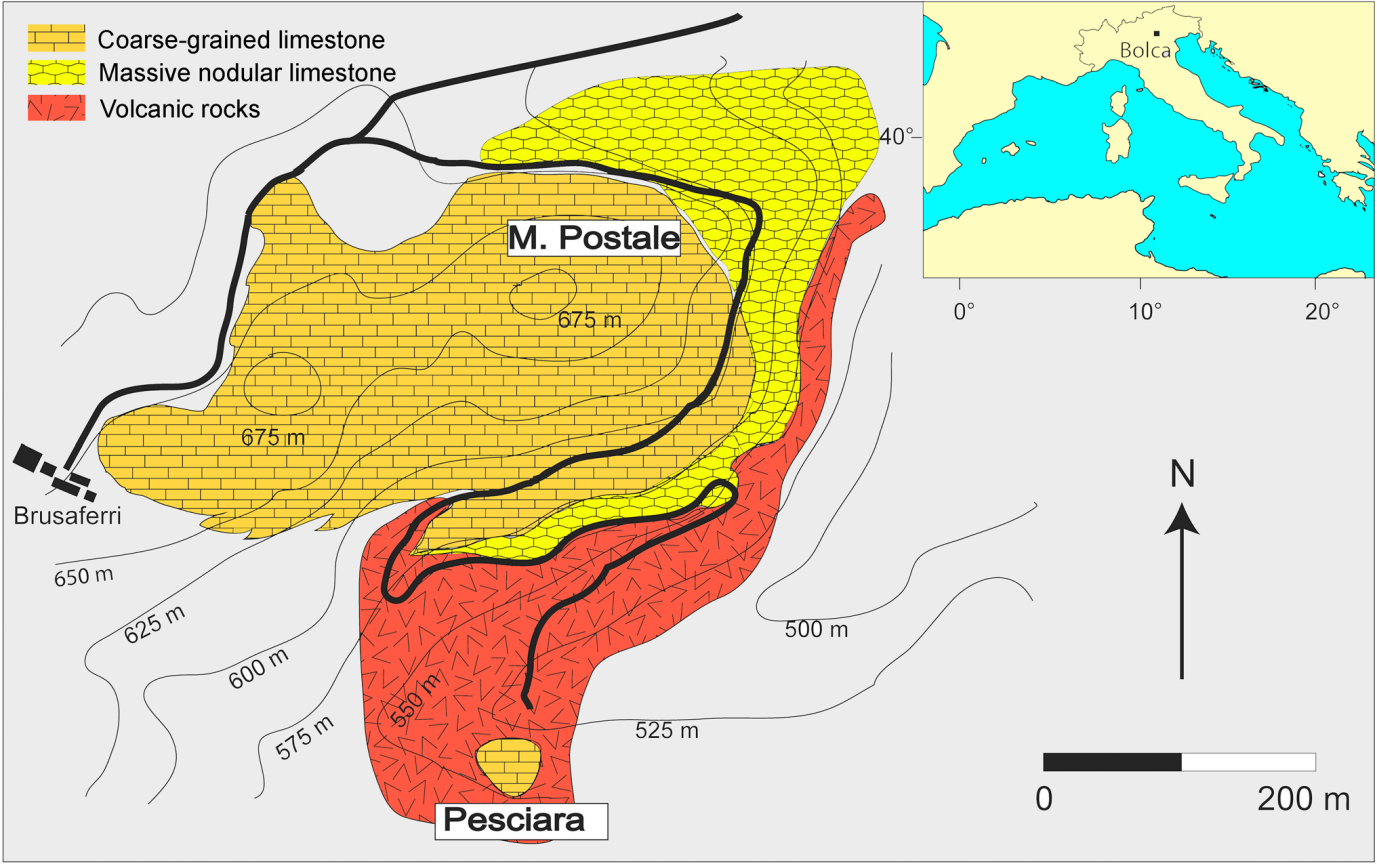
Location and schematic geological map of the Bolca area (modified from Marramà et al. 2016a)

All the chondrichthyan remains from Bolca were extracted from the fossiliferous layers of the Pesciara and Monte Postale sites, located in the eastern part of the Lessini Mountains (Southern Alps), about 2 km north-east of the village of Bolca, Verona Province, northeastern Italy (Fig. 1). These two sites are about 300 m from each other and exhibit a similar stratigraphic and sedimentological architecture, mostly related to the presence of finely laminated micritic limestone with fish and plant remains. The stratigraphic relationships between the two fossiliferous deposits was recently investigated by Papazzoni et al. (2017), who indicated that the uppermost productive sequence of Monte Postale should correlate with those of the Pesciara site, although the fossiliferous laminites of the Monte Postale appear to be slightly older (Papazzoni et al. 2017).

The stratigraphic sequence of the Pesciara site was investigated by several authors who referred the fossiliferous layers to the “Calcari Nummulitici”, an informal unit of Eocene age widely distributed in northeastern Italy (see Papazzoni and Trevisani 2006). The entire succession of the Pesciara site consists of a less than 20-m-thick cyclic alternation of finely laminated micritic limestones, with exquisitely well-preserved fishes, plants and invertebrates, and coarse-grained biocalcarenite/biocalcirudite containing a rich benthic fauna. Based on their larger benthic foraminiferan content, the fish-bearing limestones of the Pesciara site were referred to the *Alveolina dainelli* Zone or SBZ 11 Biozone (Papazzoni et al. 2014a), corresponding to the late Cuisian (late Ypresian, slightly less than 50 Ma). The controlled excavations conducted by the MCSNV between 1999 and 2011 allowed better definition of the palaeoenvironmental context of the Pesciara palaeobiotope. Results of the subsequent quantitative palaeoecological analysis by Marramà et al. (2016a) confirm that the Pesciara fish assemblage is defined by a sharp oligarchic structure dominated by the zooplanktivorous fishes (mostly clupeids), whereas the taphonomic features confirm that the sediments were deposited in an intraplatform basin in which benthic anoxic conditions and the development of a biofilm acted as promoters of the high-quality preservation of the fossils (see also Papazzoni and Trevisani 2006).

The Monte Postale succession includes the Cretaceous Scaglia Rossa Formation up to the Ypresian fossiliferous limestone. The first detailed stratigraphic study of the Monte Postale site by Fabiani (1914, 1915) assigned the entire succession to the Lutetian. More than 100 years later, the foraminiferal and the calcareous nannofossil content indicates that the uppermost productive strata of the Monte Postale site are Ypresian in age (Papazzoni et al. 2017). The palaeoecological and taphonomic study of the Monte Postale fish assemblage revealed a high diversity of fishes within a different depositional context with respect to that hypothesized for the Pesciara site (Marramà et al. 2016a). The abundance of marine and terrestrial plants, large number of invertebrates (including abundant corals) and reef-associated small-sized and juvenile fishes of the Monte Postale site indicate that the fossiliferous sediments accumulated close to an emerged coastal area (lagoon) surrounded by a coralgal rim. As such, the prominent disarticulation of fish skeletons, unimodal dispersion of the elements, and bioturbations were the results of disturbance and benthic periodic oxic conditions (Marramà et al. 2016a; Vescogni et al. 2016).

## Taxonomic remarks

Eocene chondrichthyans of Bolca include at least 17 species-level taxa belonging to 10 families, including selachians of the orders Carcharhiniformes and Lamniformes, and batoids of the orders Torpediniformes, Myliobatiformes and Rajiformes (Table 1). Included among those taxa is the first report of the occurrence of chimaeroid remains from Bolca.

**Table 1:** Synoptic list of the Eocene chondrichthyans of the Bolca Konservat-Lagerstätte

	Order	Family	Taxon
Selachi	Carchariniformes	Triakidae	*Galeorhinus cuvieri*
		Carcharinidae	*Eogaleus bolcensis*
	Lamniformes	Odontaspididae	*Brachycarcharias lerichei*
Batoidea	Torpediniformes	Narcinidae	*Titanonarke molini*
			*Titanonarke megapterygia*
	Myliobatiformes	Dasyatidae	“*Dasyatis*” *muricata*
			*“Dasyatis” zigni*
			*“Dasyatis”* sp.
		Myliobatidae	*Promyliobatis gazolae*
		Urolophidae	*“Urolophus” crassicaudatus*
			*“Urolophus”* sp.
	Rajiformes	Rhinobatidae	*“Rhinobatus” dezigni*
			*“Rhinobatus” primaevus*
		Platyrhinidae	*“Platyrhina” bolcensis*
			*“Platyrhina” gigantea*
			*“Platyrhina” egertoni*
			*“Platyrhina”* sp.
Holocephali	Chimaeriformes	Callorhynchidae	*Ischyodus* sp.

### Selachii

Sharks are represented at Bolca by members of at least two orders, the Carcharhiniformes (ground sharks) and Lamniformes (mackerel sharks). Contrary to reports by Jaekel (1894), D’Erasmo (1922) and Blot (1980), the order Orectolobiformes (bamboo sharks) is not represented in the Bolca chondrichthyan assemblage. The unique specimen housed in the CNHM, and referred by Jaekel (1894) to *Mesiteia emiliae* Gorjanovic-Kramberger, 1885, is not coming from the fossiliferous deposits of the Bolca Lagerstätte. In fact, the examination of the micropalaeontological content of the slab as well as of the presence of the clupeomorph fish *Armigatus brevissimus* suggest a Cretaceous origin for the fossil, which possibly comes from one of the famous Late Cretaceous localities of Lebanon (see Cappetta 1980a).

#### Carcharhiniformes

**Fig. 2:**
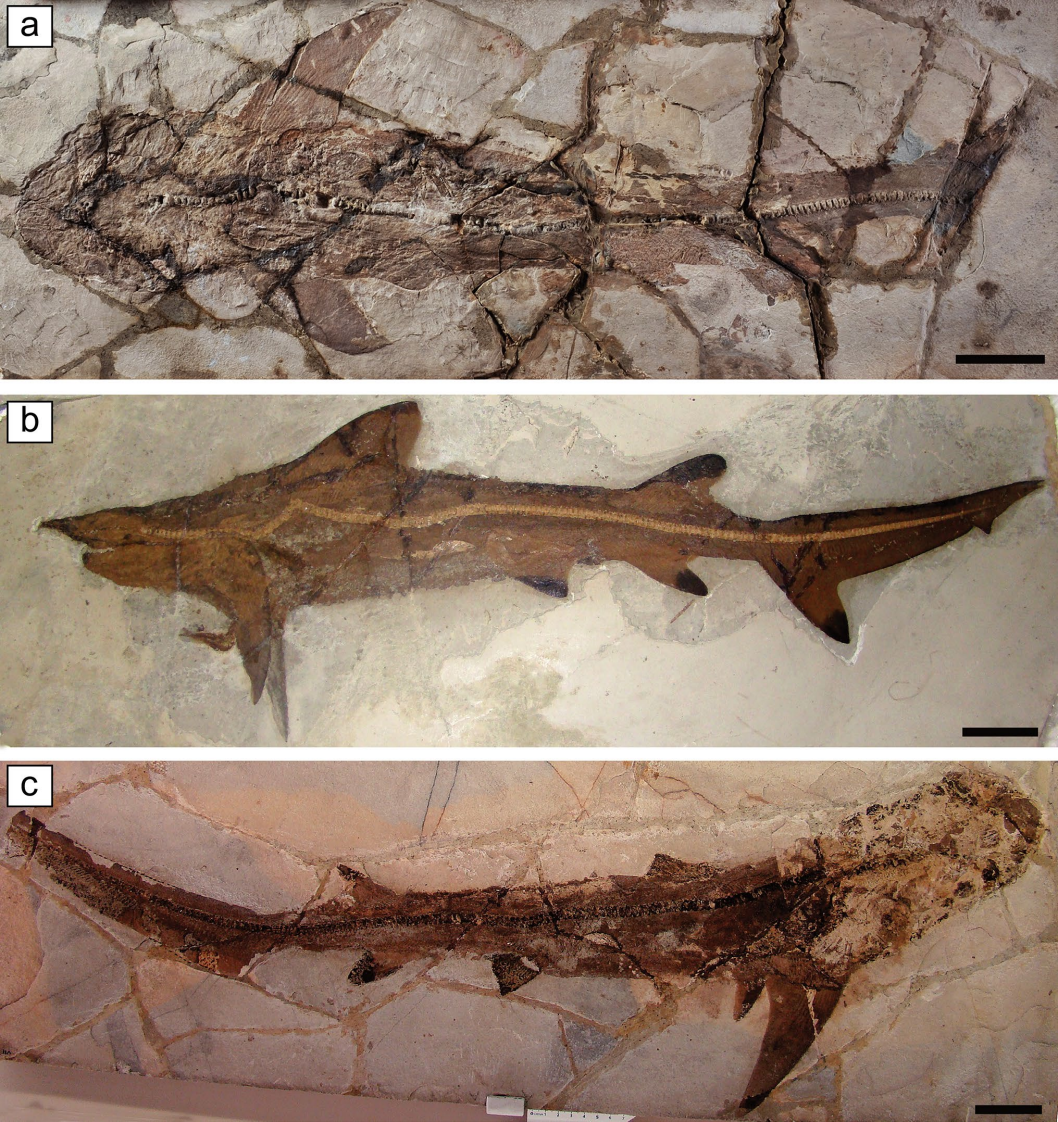
The carcharhiniform *Galeorhinus cuvieri* (Agassiz, 1835) from the Eocene Bolca Konservat-Lagerstätte: **a** MNHN F.Bol516, holotype; **b** MCSNV T.1124; **c** MCSNV VII.B.97. Scale bars 50 mm

Historically, ground sharks are among the first cartilaginous fishes from Bolca figured and described, and are currently represented by two taxa, *Galeorhinus cuvieri* (Agassiz, 1835) and *Eogaleus bolcensis* Cappetta, 1975. The taxonomic history of *Galeorhinus cuvieri* (Fig. 2) is rather complex and controversial due to the numerous studies that have attempted to define its taxonomic affinity. Volta (1796) was the first to describe and figure two specimens from the Pesciara site, referring them to the extant species *Carcharodon carcharias* (MNHN F.Bol516) and *Stegostoma fasciatus* (MCSNV VII.B.97). Later, Agassiz (1835) considered both of these specimens as conspecifics belonging to the extant genus *Galeus* and created the new species *G. cuvieri.* As additional material was described, taxonomy was again revised and Molin (1860), Lioy (1865), Jaekel (1894) and de Beaumont (1960) created additional genera (*Protogaleus*, *Alopiopsis*, *Pseudogaleus* and *Notidanus*, respectively). Eastman (1904, 1905) synonymized all these gerea later with the genus *Carcharias* (Carcharhinidae) thereby increasing taxonomic confusion. They were again synonymized within a different genus, *Galeorhinus* (Triakidae), by Cappetta (1975). A few years later, Applegate (1978) nevertheless referred these fossils to the carcharhinid genus *Alopiopsis*, which he regarded as a possible ancestor of the tiger shark *Galeocerdo*. More recently, Adnet and Cappetta (2008) suggested that this material should be assigned to the extinct carcharhinid genus *Physogaleus* (see also Cappetta 1980b). A re-examination of the holotype (MNHN F.Bol516) and additional referred specimens (MCSNV T.1124; MCSNV II.B.96/97; MCSNV B.70; MGGC 1976; MGP-PD 8871C/8872C) in 2016 has provided substantial morphological (dental and body) evidence supporting the Cappetta (1975) hypothesis and assignment to the triakid genus *Galeorhinus* (Fanti et al. 2016). Based on their results, each of the whole-bodied specimens analyzed by Fanti et al. (2016) are predicted to represent various ontogenetic stages of juvenile *G. cuvieri*, and the Pesciara palaeobiotope is hypothesized to be a nursery area for this species. Nursery areas in extant waters represent a discrete portion of a species range where gravid females congregate and usually leave their offspring, and are almost exclusively inhabited by free-swimming neonates and egg cases for most of the year (Castro 1993). Heupel et al. (2007) proposed three criteria to recognise an area as a nursery for sharks (1, sharks are more commonly encountered in the area than other areas; 2, sharks have a tendency to remain or return for extended periods; 3, the area or habitat is repeatedly used across years). In the fossil record, if shark teeth are common and depending on the variability of size among teeth collected for a given species in a specific locality, it may be possible to predict the presence of a shark nursery (Pimiento et al. 2010). Furthermore, the presence of egg cases in the fossil record provides circumstantial evidence to identify ancient shark nursery grounds (e.g., Fischer et al. 2011; Sallan and Coates 2014). None of these behavior conditions can be unquestionably tested for the Pesciara site and there is a complete absence of egg cases. Therefore, based explicitly on the occurrence of six juvenile individuals out of tens of thousands of well-preserved fossil fishes extracted from the Pesciara site, *Galeorhinus cuvieri* was an uncommon species and is thus incompatible with the requirement of high number of individuals for the identification of a nursery area (see also Marramà et al. 2017b).

Although the fossil record of *Galeorhinus* extends back to the Cenomanian (Popov and Lapkin 2000) and this genus is represented by at least 15 species from the Upper Cretaceous to Pleistocene of Europe, North and Central America, and North Africa (Adnet and Cappetta 2008; Cappetta 2012), *G. cuvieri* is the only fossil species of the genus known by completely articulated skeletons. The genus *Galeorhinus* is well represented in the Eocene of Europe by three other Ypresian species (*G. duchaussoisi*, *G. louisi* and *G. ypresiensis*), which differ from each other and from the Bolca species by having a different tooth morphology (Adnet and Cappetta 2008). The existence of at least four coeval *Galeorhinus* species in the Ypresian of Europe supports the hypothesis that the representatives of the family Triakidae were probably more diverse than today (with *G. galeus* as the only extant representative of the genus), and that the presently reduced geographic and taxonomic diversity of *Galeorhinus* represents a recent phenomenon (Adnet and Cappetta 2008).

**Fig. 3:**
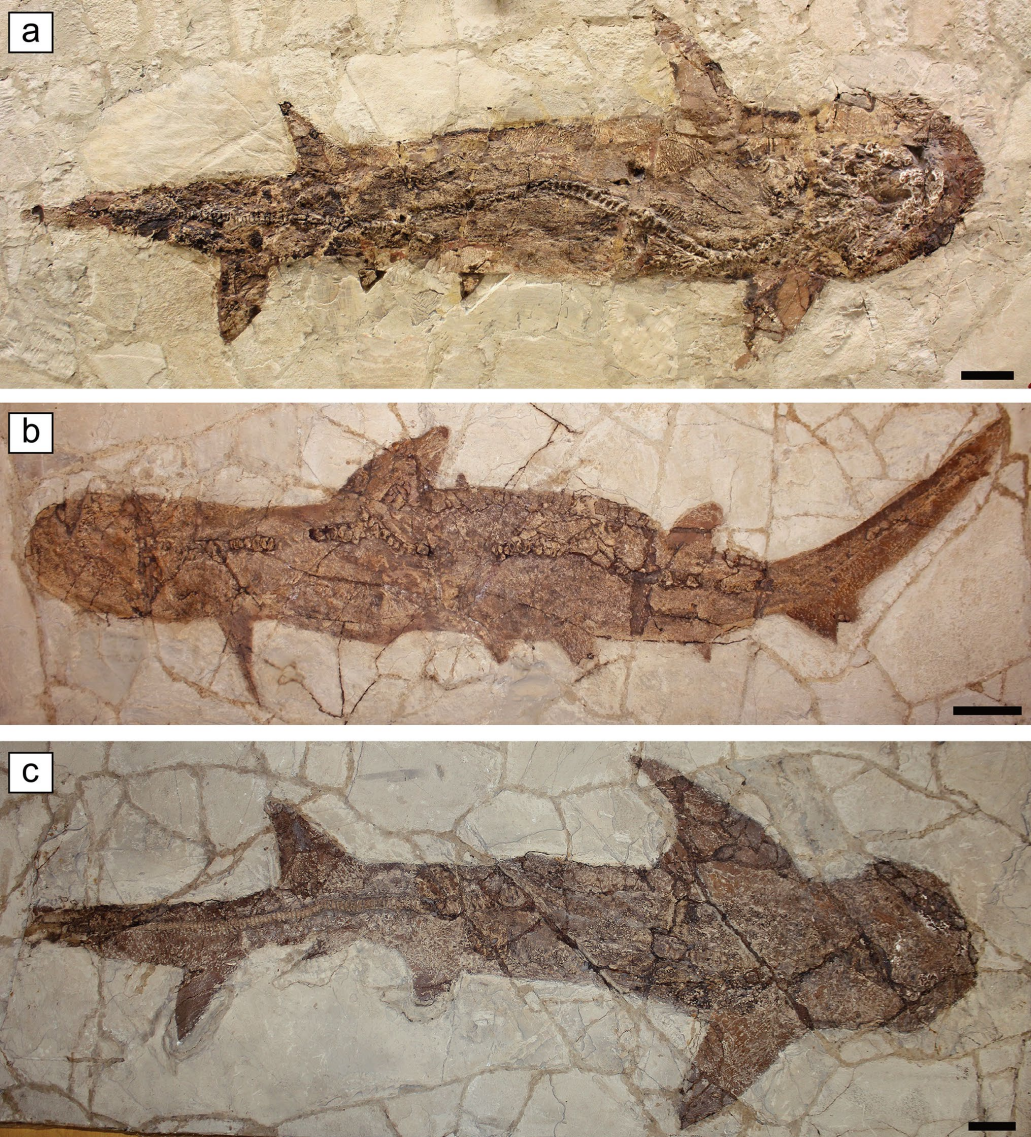
The carcharhiniform *Eogaleus bolcensis* Cappetta, 1975 from the Eocene Bolca Konservat-Lagerstätte: **a** MCSNV T.331, holotype; **b** MGP-PD 8869C/8870C; **c** MCSNV VII.B.94. Scale bars 50 mm

A second carcharhiniform (Fig. 3), *Eogaleus bolcensis* (Carcharhinidae), was described by Cappetta (1975) and clearly differs from *G. cuvieri* by having different body proportions and tooth size and morphology (Cappetta 1975; Fanti et al. 2016). According to Cappetta (1975), this taxon is based on three articulated specimens, MCSNV T.331 (holotype), MGP-PD 8869C/8870C and MCSNV VII.B.94. The latter was formerly referred to *Alopiopsis plejodon* by Jaekel (1894, pl. 8). Applegate (1978) considered *G. cuvieri* and *E. bolcensis* to be synonymous and referred them to the extinct genus *Alopiopsis*, created by Lioy (1865) based on a single specimen deposited in Vicenza, Italy. Its type species, *Alopiopsis plejodon*, was destroyed during the Second World War (Cappetta 1975; Blot 1980) and the original illustrations of Lioy (1865) are unclear and difficult to interpret; thus, the validity and taxonomic affinities of *A. plejodon* are impossible to establish [see Cappetta (1975) for more information about the taxonomic history of the genus *Alopiopsis* Lioy, 1865]. In any case, a detailed redescription of *Eogaleus bolcensis* (which is out of the scope of the present study) is necessary in order to definitively assess its taxonomic placement as well as to confirm the presence of the family Carcharhinidae in the Bolca fish assemblage.

Lithological and sedimentological evidences seem to suggest that the skeletal material of the carcharhiniform species *G. cuvieri* and *E. bolcensis* derive from the Pesciara site (see also Cappetta 1975; Fanti et al. 2016).

#### Lamniformes

According to the synoptic list of the Eocene chondrichthyans from Bolca provided by Blot (1980), the sharks were represented only by the orders Orectolobiformes and Carcharhiniformes. However, as noted earlier, the orectolobiform shark, *Mesiteia emiliae*, was not actually from Bolca. Furthermore, Blot (1980) did not consider the isolated lamniform teeth from the Monte Postale site described by Bassani (1897), and those teeth derived from subsequent excavations in both sites.

**Fig. 4:**
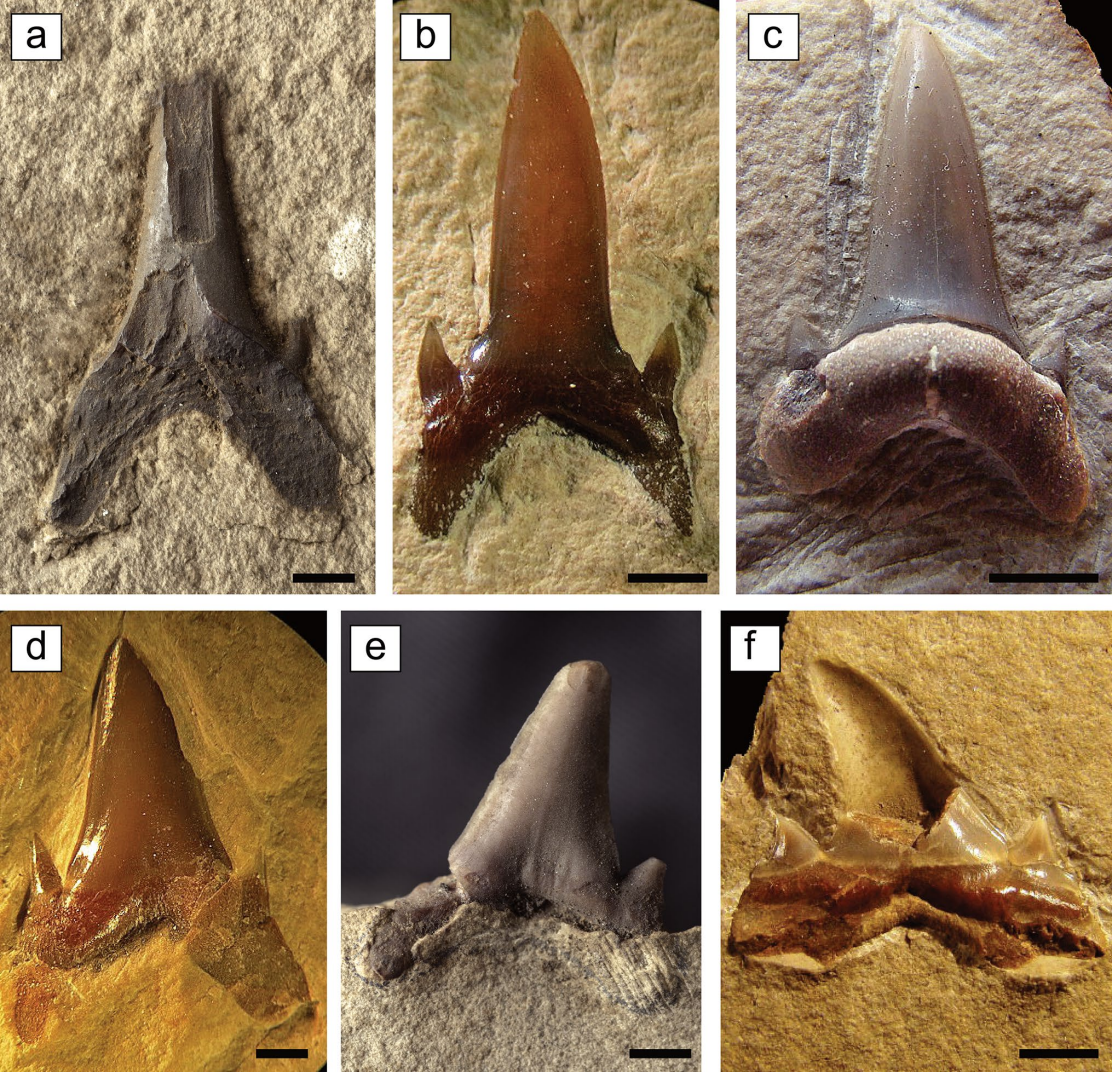
Isolated teeth of the sand tiger shark *Brachycarcharias lerichei* (Casier, 1946) of the order Lamniformes from the Eocene Bolca Konservat-Lagerstätte. Anterior teeth: **a** MGP-PD 7358, lingual view; **b** MCSNV IG.VR.69800, labial view; lower antero-lateral teeth: **c** MCSNV IG.135779, lingual view; **d** MCSNV IG.VR.66977, labial view; upper lateral teeth: **e** MGP-PD 7366, labial view; **f** MCSNV T.176, labial view. Scale bars 2 mm

Bassani (1897) reported some teeth referred to *Lamna vincenti* Winkler, 1874 (Fig. 4). The presence of this species was reported in several Eocene (Ypresian) deposits of Europe, North America and North Africa (e.g., Woodward 1899; Casier 1946; Arambourg 1952; Noubhani and Cappetta 1997). In the first revision of the Ypresian material from Belgium, specimens traditionally assigned to *L*. *vincenti* were reclassified as *Lamna lerichei* by Casier (1946). In a later revision of the Eocene odontaspidid material from Belgium by Cappetta and Nolf (2005), the *Lamna lerichei* teeth were observed to be extremely morphologically different from those of any known odontaspidid or lamnid species. Thus, they erected the odontaspidid taxon *Brachycarcharias lerichei*, which included *Lamna vincenti* and *L*. *lerichei*. Based on a recent revision of the lamniform material from Pesciara and Monte Postales sites (Marramà et al. 2017b), teeth described and figured by Bassani (1897) as *L. vincenti* (Fig. 4a, e), and those collected from the recent 1999–2011 controlled excavations correspond perfectly to the diagnosis of the anterior and lateral teeth of *Brachycarcharias lerichei* (Casier, 1946), a species widely spread across the Northern Hemisphere during the early Paleogene.

Of note is a unique, large (about 50 mm) shark tooth (today housed in the MGP-PD) figured in “Ittiolitologia Veronese” (pl. 3, Fig. 2), which, it was clearly stated, did not derive from Bolca (Volta 1796). However, Agassiz (1835) created the species *Carcharias sulcidens* for this specimen and included it in the list of the fishes of Bolca. De Zigno (1874a) discussed interpretations about the systematic position and provenance of this specimen and subsequently assigned it to the genus *Carcharodon*, and indicated its Bolca origin as questionable. Bassani (1897) and D’Erasmo (1922) agreed with this latter hypothesis, and referred the specimen to *Carcharodon auriculatus* (de Blainville, 1818). Schauroth (1865) referred a different isolated tooth housed in the Cobourg museum in Germany (see de Zigno 1874a) to *Otodus macrotus* Agassiz, 1833–1844, indicating Bolca as the locality of provenance. However, its origin and taxonomic placement are also uncertain.

Finally, the abundant isolated odontaspidid teeth historically extracted from the Spilecco site, another fossiliferous quarry near the Bolca area, and referred by Bassani (1897) to *Odontaspis hopei* (see also Marramà et al. 2017b), are not considered in this review since the sedimentological and stratigraphic features, as well as the age, the palecological and palaeoenvironmental context, are remarkably different from those of the Pesciara and Monte Postale sites (see Papazzoni et al. 2014b).

### Batoidea

Skates and rays (Batoidea) are the most diverse group of Eocene chondrichthyans in the Bolca Lagerstätte, represented by members of the orders Torpediniformes, Rajiformes and Myliobatiformes. Although several attempts have been devoted to resolving the phylogeny of batoid fishes based on molecular and morphological features (e.g., McEachran et al. 1996; McEachran and Aschliman 2004; Aschliman et al. 2012a, b; Claeson et al. 2013), their relationships remain controversial. A comprehensive revision of the poorly known batoids from Bolca would contribute to resolving this problematic issue.

#### Torpediniformes

**Fig. 5:**
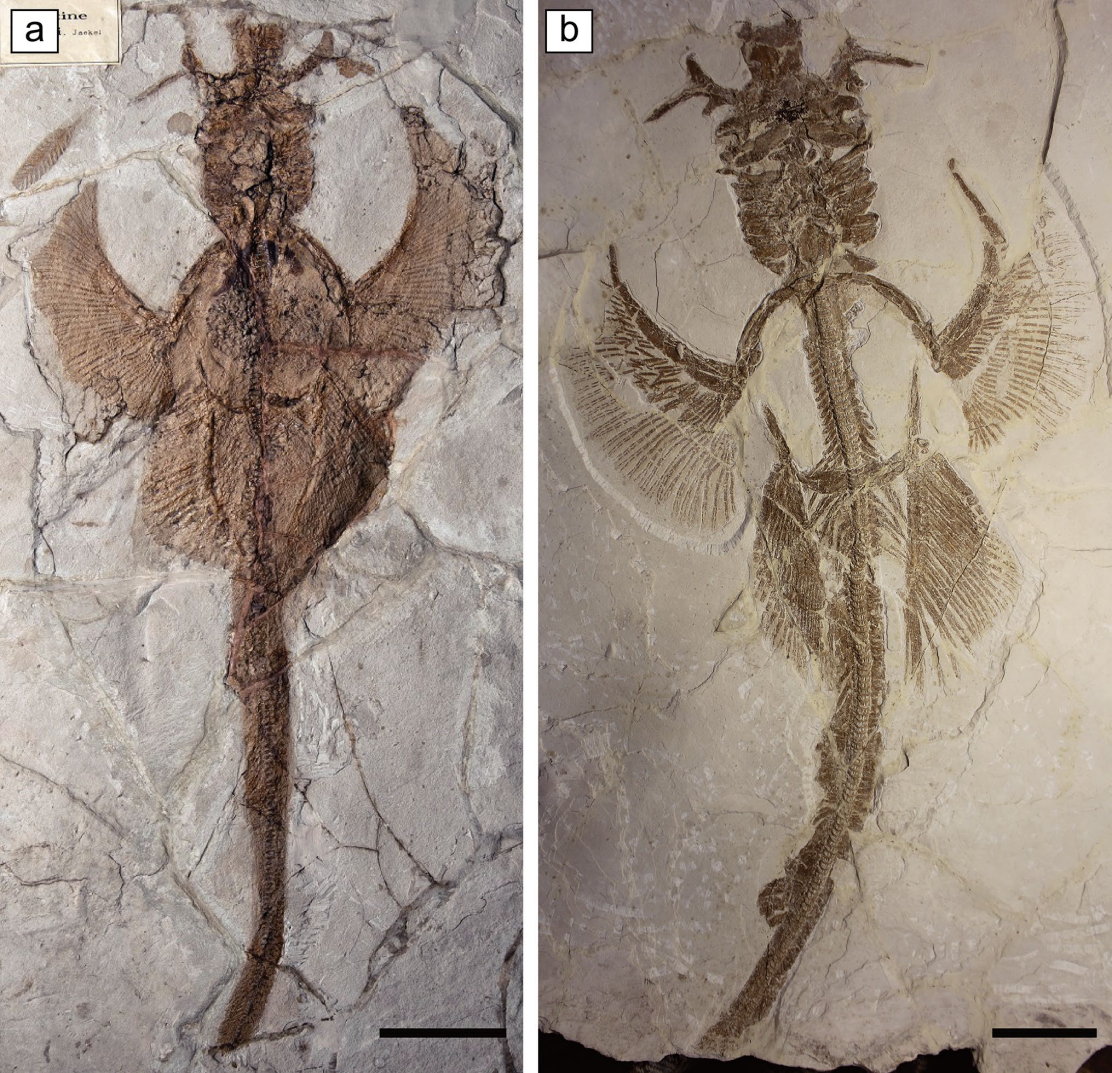
The numbfish *Titanonarke molini* (Jaekel, 1894) of the order Torpediniformes from the Eocene Bolca Konservat-Lagerstätte: **a** MGP-PD 26275, holotype; **b** MCSNV IG.VR.67290. Scale bars 100 mm

**Fig. 6:**
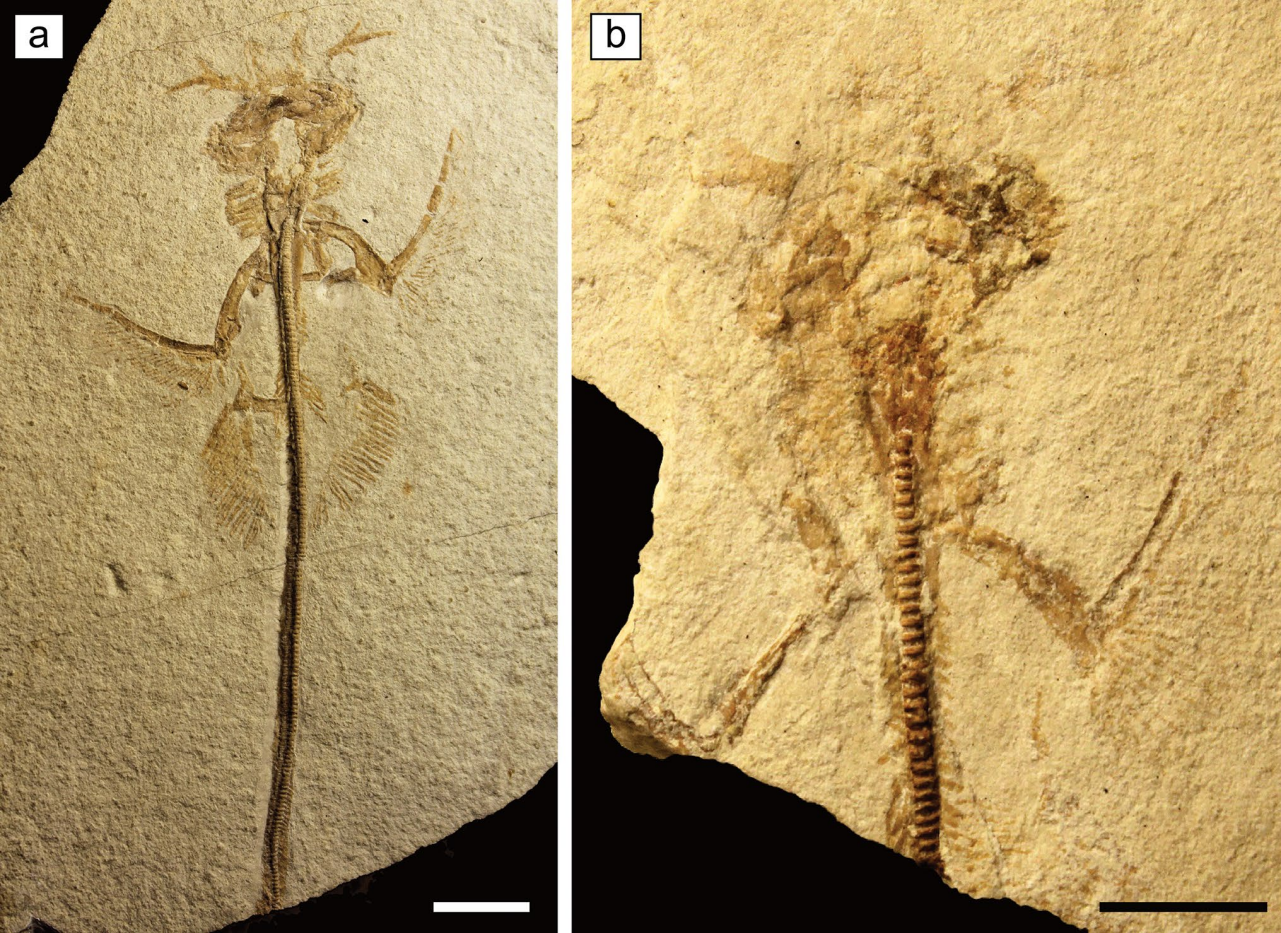
Juvenile individuals of *Titanonarke molini* (Jaekel, 1894) from the Eocene Bolca Konservat-Lagerstätte: **a** MCSNV IG.VR.91359; **b** MCSNV IG.135581. Scale bars 10 mm

Electric rays of the order Torpediniformes can be distinguished from all the other batoids mainly by the presence of massive electric organs that develop between the axial and pectoral skeleton, anteriorly directed, fan- or antler-shaped antorbital cartilages, and lack of dermal denticles (Compagno 1999; Claeson 2014). Torpediniforms are currently represented at Bolca by the genus *Titanonarke* (Narcinidae) by de Carvalho (2010), containing the species *Narcine molini*, which was created by Jaekel (1894) based on the holotypic specimen MGP-PD 25275/6. This species is known from numerous individuals representing different ontogenetic stages from the Monte Postale site (Figs. 5, 6). *Titanonarke* is by far the physically largest (up to1 m) representative of the non-torpedinid Torpediniformes (Narcinoidei). However the diagnosis of this genus provided by de Carvalho (2010) is somewhat problematic. For instance, taphonomic biases cannot be excluded to explain the apparent absence of typical narcinid characters considered diagnostic of *Titanonarke* (e.g., absence of dorsal fins and of the posteriorly directed branches of the antorbital cartilages; see de Carvalho 2010). A detailed revision of the known *Titanonarke* material and an integrated comprehensive phylogenetic analysis clarified several aspects of the anatomy and systematics of the Bolca electric rays and their systematic position within the Torpediniformes (Marramà et al. 2017a).

Although fossil remains of additional torpediniforms are known from the early Paleogene of Europe, North Africa and North America (see Cappetta 2012), *Titanonarke* is the only electric ray represented by complete articulated skeletons. Other Eocene torpediniforms referred to *Eotorpedo* and to the extant genera *Torpedo* and *Narcine* are represented by isolated teeth known from Africa (Cappetta 1987, 1988), Near East (Cappetta et al. 2000), France and Belgium (Adnet 2006) and North America (Case et al. 2015).

#### Myliobatiformes

Stingrays of the order Myliobatiformes are the most diverse batoids of the Bolca fish assemblage and include at least six species-level taxa in three families (Dasyatidae, Urolophidae, Myliobatidae). All known myliobatiforms from Bolca were considered in detail by Jaekel (1894) and warrant new morphological and phylogenetic analyses to properly interpret the structure and affinities of these extraordinary fossils (see also de Carvalho et al. 2004).

**Fig. 7:**
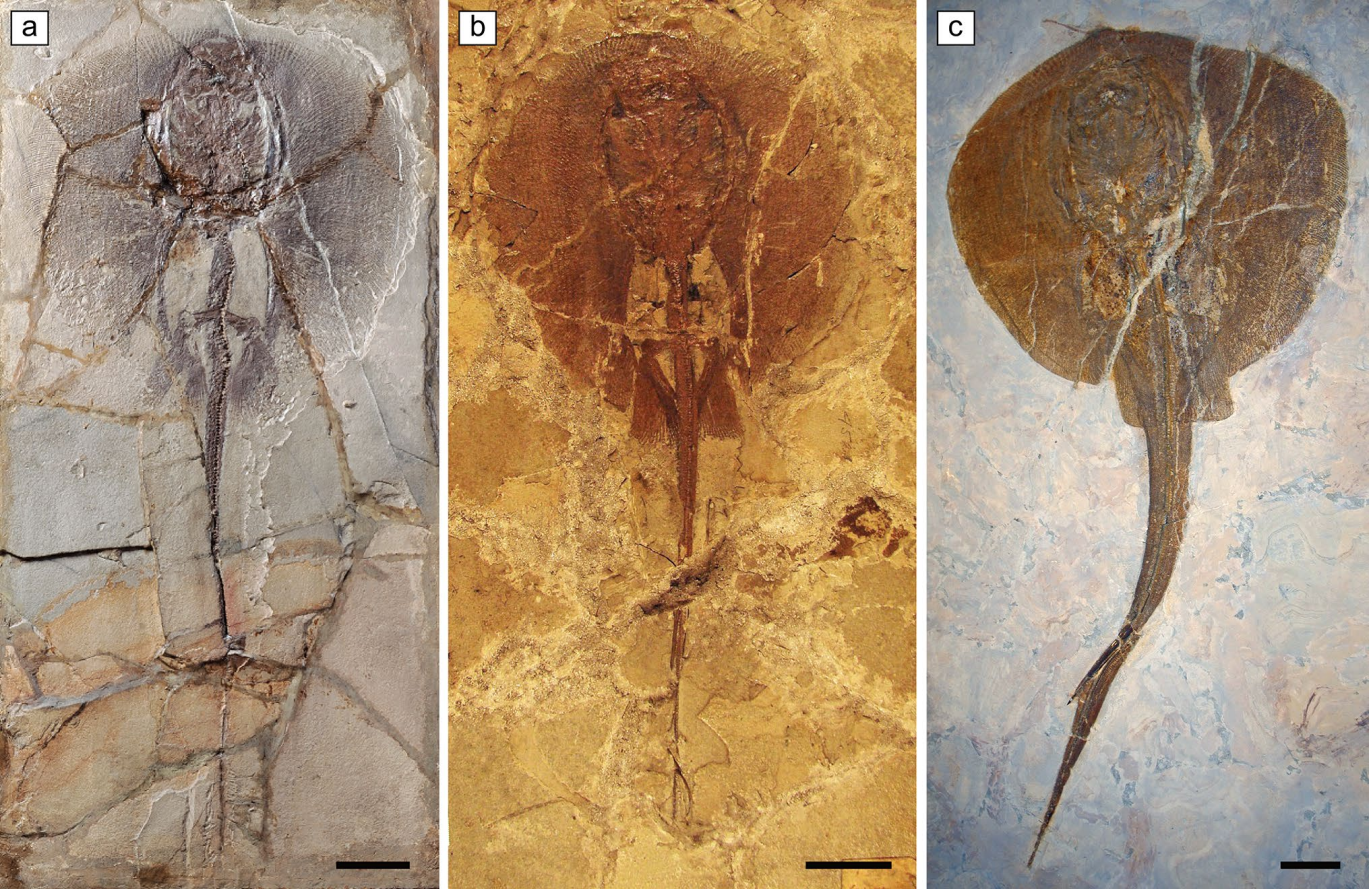
The whiptail stingray “*Dasyatis*” *muricata* (Volta, 1796) of the family Dasyatidae from the Eocene Bolca Konservat-Lagerstätte: **a** MNHN F.Bol564, holotype; **b** CM 4521; **c** MSNM V714. Scale bars 50 mm

**Fig. 8:**
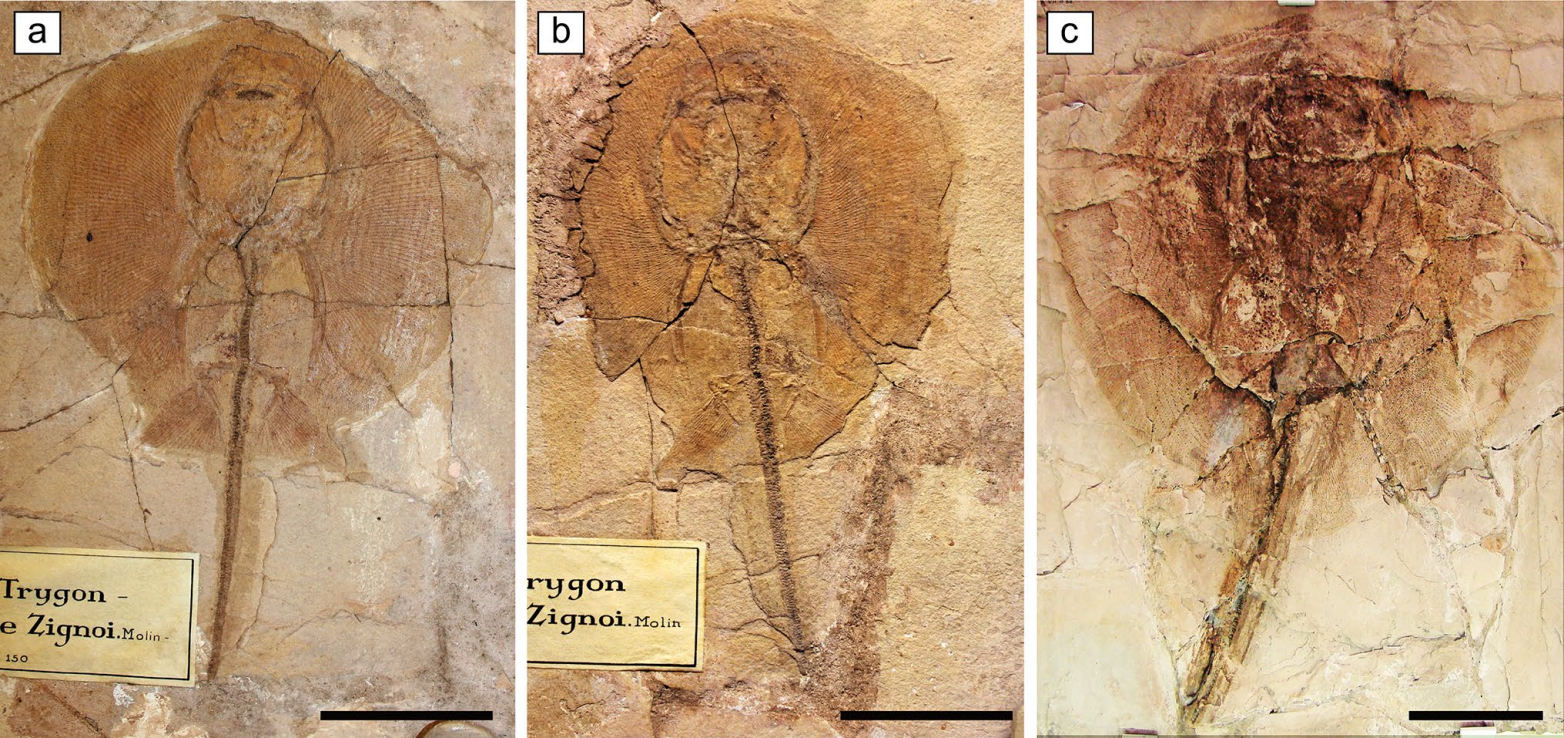
The whiptail stingray “*Dasyatis*” *zignii* (Molin, 1861) from the Eocene Bolca Konservat-Lagerstätte: **a**, **b** MGP-PD 150Z/151Z, holotype in part and counterpart; **c** MCSNV VII.B.87. Scale bars 50 mm

Whiptail stingrays of the family Dasyatidae, characterised by a disc no more than 1.3 times as broad as long, a long tail without dorsal and caudal fins, and one or more long poisonous stings (Compagno 1999; Nelson 2006; Cappetta 2012), are represented at Bolca by two species (Figs. 7, 8). “*Dasyatis*” *muricata* is one of the first cartilaginous fishes described from Bolca, represented by at least a dozen specimens and characterised by a controversial taxonomic history. This taxon was described and figured originally by Volta (1796) under the name *Raja muricata* based on an exquisitely well-preserved specimen in part and counterpart currently housed in the MNHN (F.Bol564, holotype, Fig. 7a). Since then, it has had a complex and intricate taxonomic scenario. As reviewed by Woodward (1889), the holotype was reassigned to *Trygonobatis vulgaris* and *Trygon gazolae* by de Blainville (1818) and Agassiz (1835, 1833–1844), respectively. *Taeniura knerii* was then erected by Molin (1861), and specified as *Alexandrinum molinii* based on some additional material by de Zigno (1874b). These taxa and their respective specimens were then synonymized as *Trygon* Cuvier 1816, by Jaekel (1894), where *Raja muricata* Volta, 1796, was the holotype. After examining the Bayet collection of Bolca specimens which were accessioned into the CM in 1903 (e.g., CM 4521, CM 4304; Fig. 7b), the synonymy of *Trygon muricata* was upheld by Eastman (1904, 1905, 1911), with the exception of a specimen of *Taeniura knerii* which was considered a synonym of *Urolophus crassicaudatus.*

Molin (1861) described another dasyatid species as *Anacanthus zigni* based on a single specimen deposited in the MGP-PD (150Z/151Z; Fig. 8a, b; see also de Zigno 1874a, b). Subsequently, Jaekel (1894) assigned it to the genus *Trygon* in his review of the Bolca myliobatiforms as well. At present, *Trygon* is regarded as a junior synonym of *Dasyatis* Rafinesque 1810, thus we refer to these species as “*Dasyatis*” *muricata* and “*D.*” *zigni*.

De Carvalho et al. (2004) hypothesized a systematic placement of “*Dasyatis*” *muricata*, thus assuming close relationships to extant members of the family Dasyatidae in their study of Eocene stingrays from the North America Green River Formation. The genus *Dasyatis*, nevertheless, is seemingly polyphyletic according to a morphologically based phylogenetic analysis of extant whiptail stingray species by Rosenberger (2001). So far, more than 70 nominal fossil species have been described, based predominantly on isolated teeth from the Early Cretaceous (Hauterivian, although the first species close to modern forms is probably Maastrichtian) to Pliocene (Zanclean) of Europe, Africa, North America and Asia (see Cappetta 2012). However, although Cappetta (2012) did not find any notable differences in the skeletal anatomy between extant members of the genus *Dasyatis* and thus assigned these specimens from Bolca tentatively to this genus, an updated comparative analysis is urgently needed to establish the taxonomic diversity of whiptail stingrays in the Bolca sites.

**Fig. 9:**
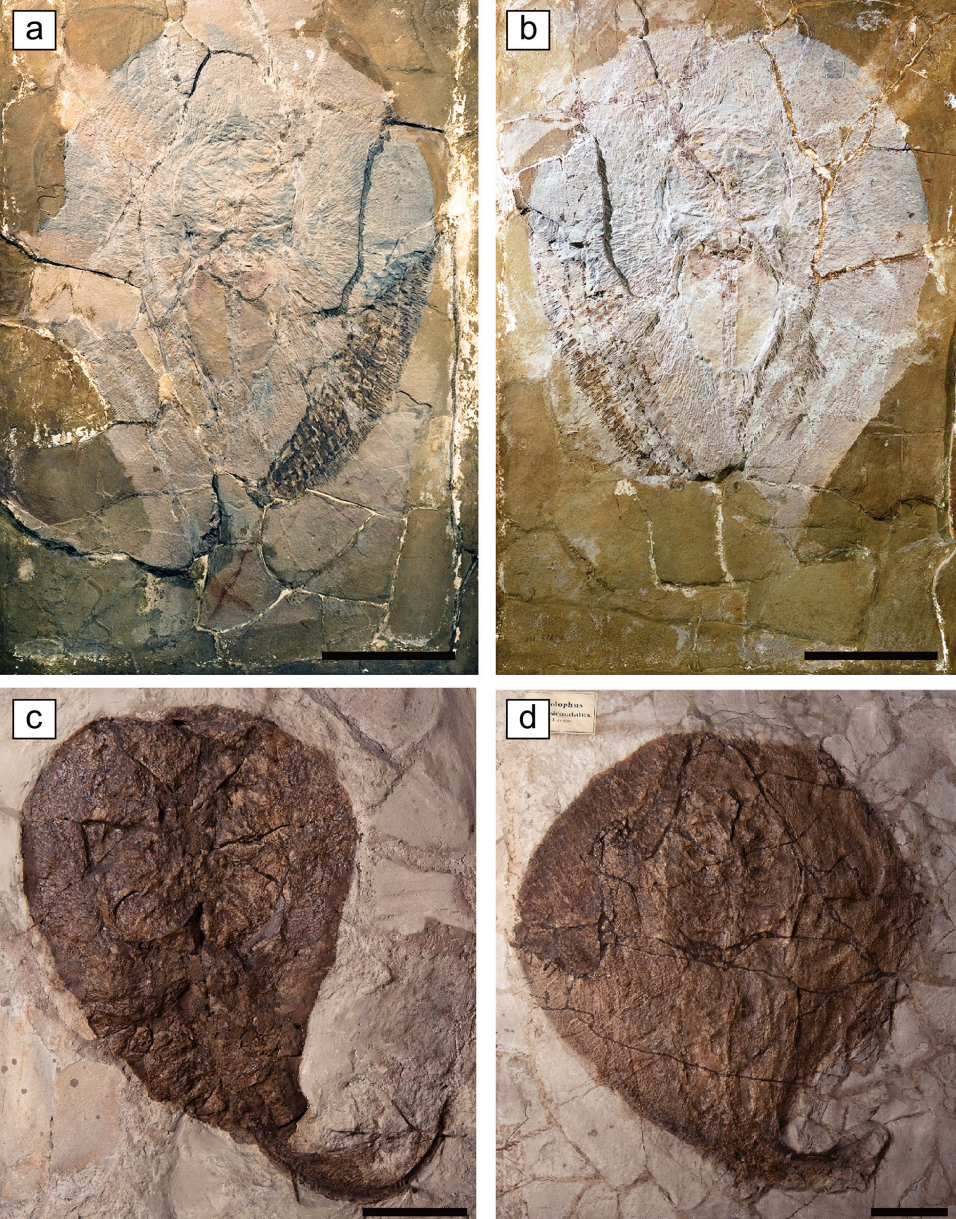
The round stingray “*Urolophus*” *crassicaudatus* (de Blainville, 1818) of the family Urolophidae from the Eocene Bolca Konservat-Lagerstätte: **a**, **b** MNHN F.Bol566, holotype in part and counterpart; **c** MGP-PD 26277; **d** MGP-PD 8875C. Scale bars 100 mm

Urolophids, also known as round stingrays or stingarees, are characterised by a disc less than 1.3 times as broad as long (as in dasyatids), the presence of a dorsal fin usually and a tail that is moderately long with a barbed spine and caudal fin (Compagno 1999; Nelson 2006; Cappetta 2012). De Blainville (1818) described a single specimen, which is housed in the collection of the MNHN (F.Bol566; see Fig. 9a, b), as *Trygonobatus crassicauda* that subsequently was transferred to *Trygon oblongus* by Agassiz (Agassiz 1835, 1833–1844; see also de Zigno 1874a). Another specimen housed in the NHMW was referred to *Urolophus princeps* by Heckel (1853), and this taxonomic assignment was later maintained by de Zigno (1874a). Jaekel (1894) provided a re-description of both these specimens together with additional other material deposited in the MGP-PD and MCSNV (Fig. 9c, d), and concluded that *U. princeps* should be regarded as a junior synonym of *T. crassicauda*. Consequently, he referred all known material to the genus *Urolophus*. Eastman (1904, 1905), D’Erasmo (1922) and Blot (1980) followed this latter taxonomic placement. Additional specimens collected since that time also were identified as “*Urolophus*” sp. by de Carvalho et al. (2004). These specimens from Bolca represent the only fossil articulated skeletal urolophid remains known to date. According to Cappetta (2012), the size and morphology of the teeth of “*U*.” *crassicaudatus* are quite different from those of extant *Urolophus* species, being morphologically similar to those of the genus *Arechia* Cappetta, 1983.

**Fig. 10:**
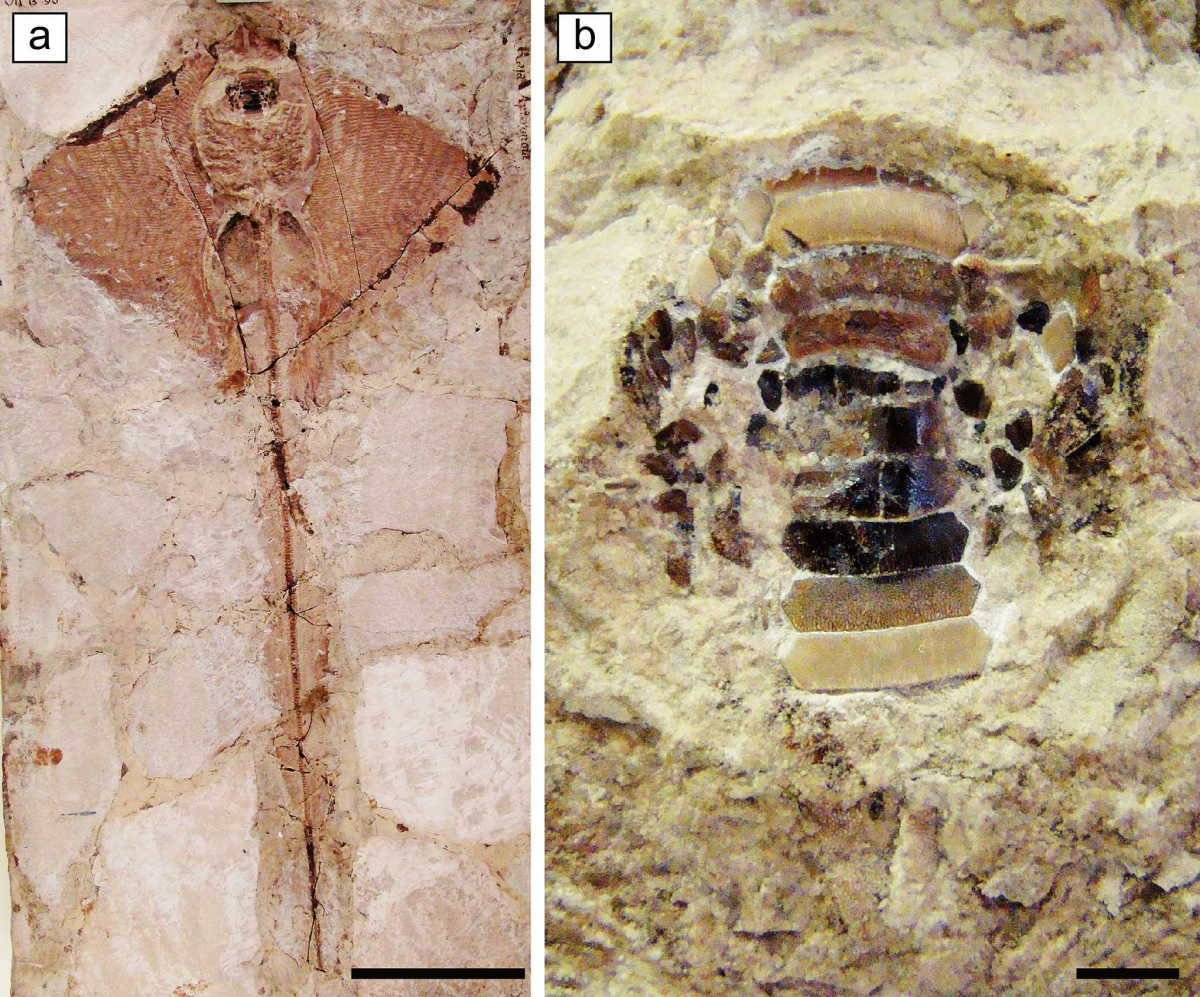
The eagle ray *Promyliobatis gazolae* (de Zigno, 1885) of the family Myliobatidae from the Eocene Bolca Konservat-Lagerstätte: **a** MCSNV VII.B.90, holotype; scale bar 100 mm; **b** Close up of the oral region of the same specimen showing the typical tooth pattern of the myliobatids; scale bar 10 mm

Eagle rays are represented at Bolca by a single completely articulated skeleton in part and counterpart (MCSNV VII.B.90/91; Fig. 10a) showing the typical characters of the family Myliobatidae, including thick rostral fins, large flat-crowned teeth with a hexagonal occlusal outline forming distinctive crushing plates in which the medial teeth are very wide (see Fig. 10b), a long tail with a functional sting, and no caudal fin (see Compagno 1999; Cappetta 2012). The fossil specimen originally was described by de Zigno (1885) as *Myliobates gazolai,* then re-described by Jaekel (1894) who, highlighting the differences with the extant *Myliobatis*, transferred the specimen to the genus *Promyliobatis*. A detailed morphological and phylogenetic analysis of this fossil again is in urgent need of revision.

The site of origin of the skeletal material referred to myliobatiforms appears to be difficult to define. The lithology of the slabs that include the skeletal material, as well as its high- quality preservation, suggests that most of the specimens derive from the Pesciara site, even if origination from the Monte Postale site currently cannot be definitively excluded for a few specimens (see e.g., Heckel 1853; de Zigno 1874a).

#### Rajiformes

Non-torpediniform and non-myliobatid batoids at Bolca currently comprise “Rhinobatidae” (guitarfishes) and Platyrhinidae (thornbacks), whose taxonomic placement remains controversial. Some authors included guitarfishes and thornbacks within the Rhinobatiformes (Compagno 1999), or, alternatively, assigned them to the Pristiformes and Myliobatiformes, respectively (see Nelson 2006; Nelson et al. 2016). However, the most recent phylogenetic analysis based on morphological characters to include extinct and extant “rhinobatids” and platyrhinids recovers them within the clade Rajiformes, where Rajiformes and Myliobatiformes are more closely related to each other than either is to Torpediniformes (Claeson et al. 2013).

**Fig. 11:**
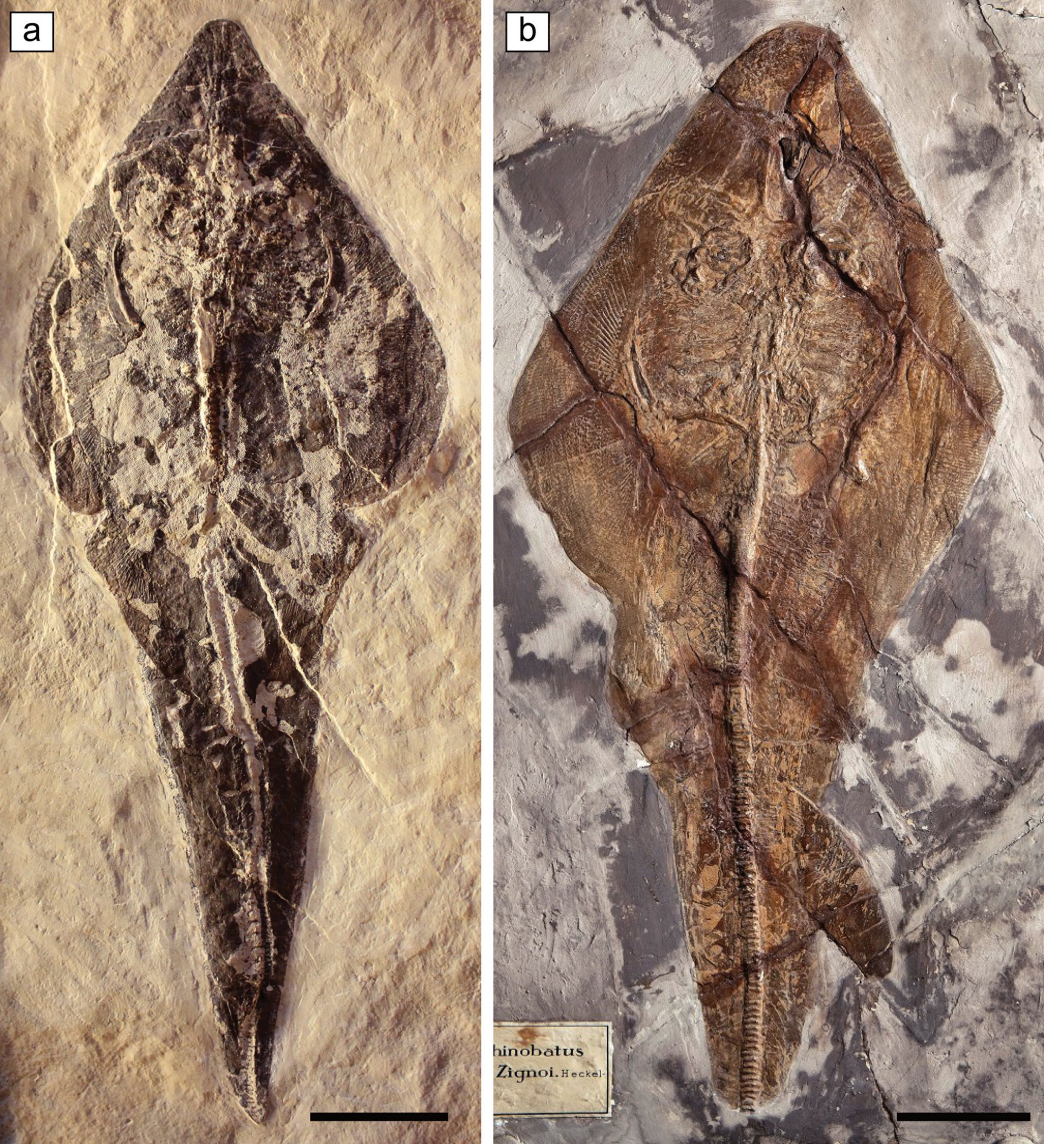
Guitarfishes of the family “Rhinobatidae” from the Eocene Bolca Konservat-Lagerstätte: **a** “*Rhinobatus*” *dezigni* (Heckel, 1853), NHMW 1853.XXVII.4, holotype; **b** “*Rhinobatus*” *primaevus* de Zigno, 1874a, MGP-PD 26278, holotype. Scale bars 100 mm

Typical “rhinobatid” batoids are characterised by an angular snout, large angular or rounded pectoral fins forming a wedge-shaped disk with head, small thorns around the eyes, shoulder and midline of the body, two dorsal fins with the first dorsal fin origin behind the pelvic rear tips, and pelvic fins not notched (Compagno 1999; Cappetta 2012). The first description of guitarfishes from Bolca was provided by Heckel (1853) who created the taxon *Trygonorhina dezignii* based on a single exquisite specimen from the Monte Postale site, currently housed in the NHMW (1853.XXVII.4; Fig. 11a). Later, the specimen was briefly redescribed by de Zigno (1874a) and included within the extant genus “*Rhinobatus*” (=*Rhinobatos*) by Jaekel (1894). The second rhinobatid species from Bolca was described de Zigno (1874a, 1876) and Jaekel (1894) as *Rhinobatus primaevus* based on a single specimen from Monte Postale in the collection of the MGP-PD (26278; Fig. 11b). Both “*R*.” *dezignii* and “*R.*” *primaevus* subsequently were included in the catalogues of the fishes from Bolca published by Eastman (1904, 1905), D’Erasmo (1922) and Blot (1980). A modern detailed morphological and phylogenetic analysis of these Eocene “rhinobatids” is certainly necessary.

Several fossils of guitarfishes were traditionally referred to the extant genus *Rhinobatos* based on articulated specimens or isolated teeth ranging from the Lower Cretaceous (Barremian) to the Pliocene (Zanclean) of Europe, Africa, North and South America and Asia (see Kriwet et al. 2007; Cappetta 2012). However, as shown by phylogenetic studies of Cretaceous species of Gondwanan “*Rhinobatos*” (Brito and Dutheil 2004; Claeson et al. 2013), the species assigned to that genus do not constitute a monophyletic group. A revision of modern and fossil species (including the Monte Postale specimens) might clarify the relationships among species of “*Rhinobatos*”.

**Fig. 12:**
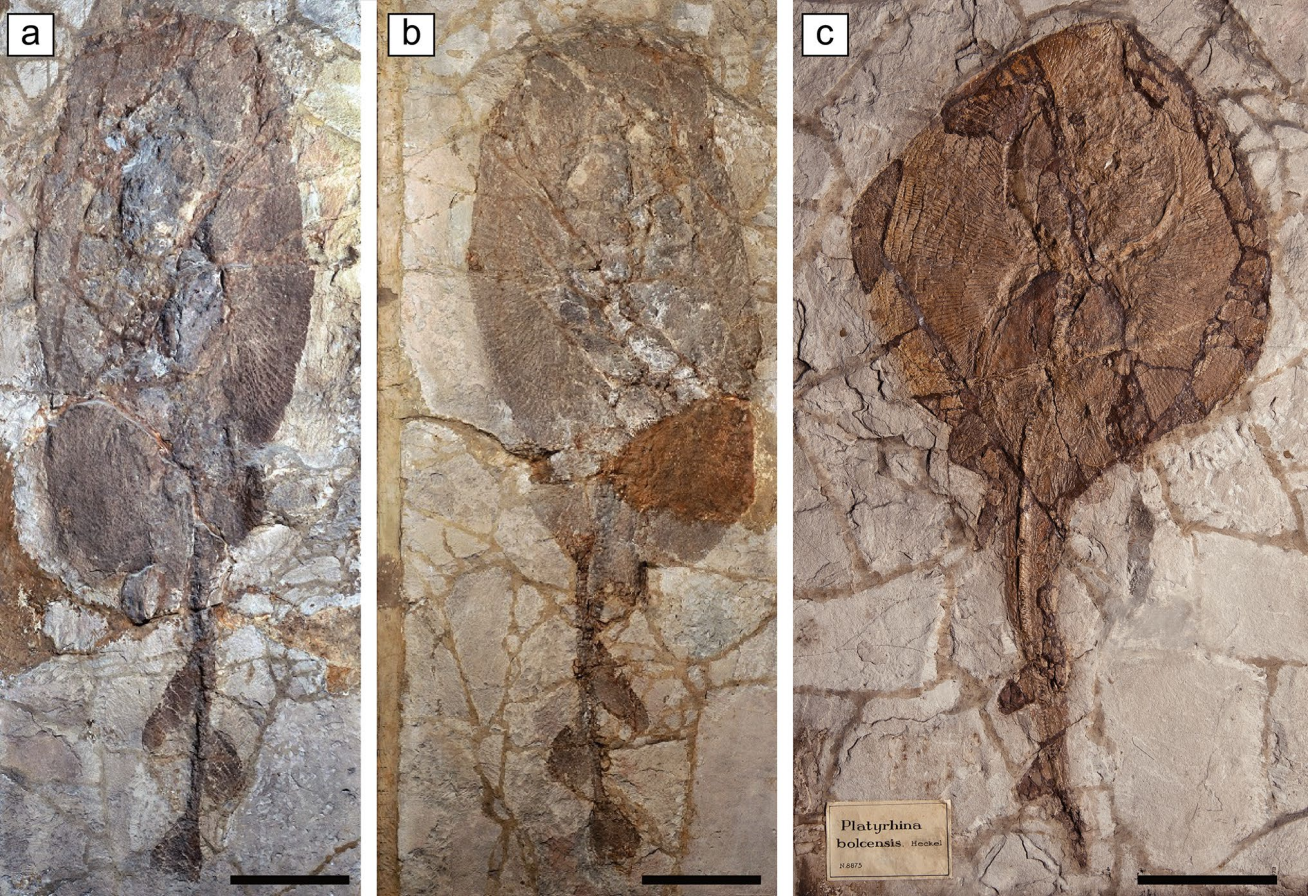
Thornbacks of the family Platyrhinidae from the Eocene Bolca Konservat-Lagerstätte: **a, b** “*Platyrhina*” *gigantea* (de Blainville, 1818), MNHN F.Bol567, holotype in part and counterpart; **c** “*Platyrhina*” *bolcensis* Heckel, 1851, MGP-PD 8875C, holotype. Scale bars 100 mm

**Fig. 13:**
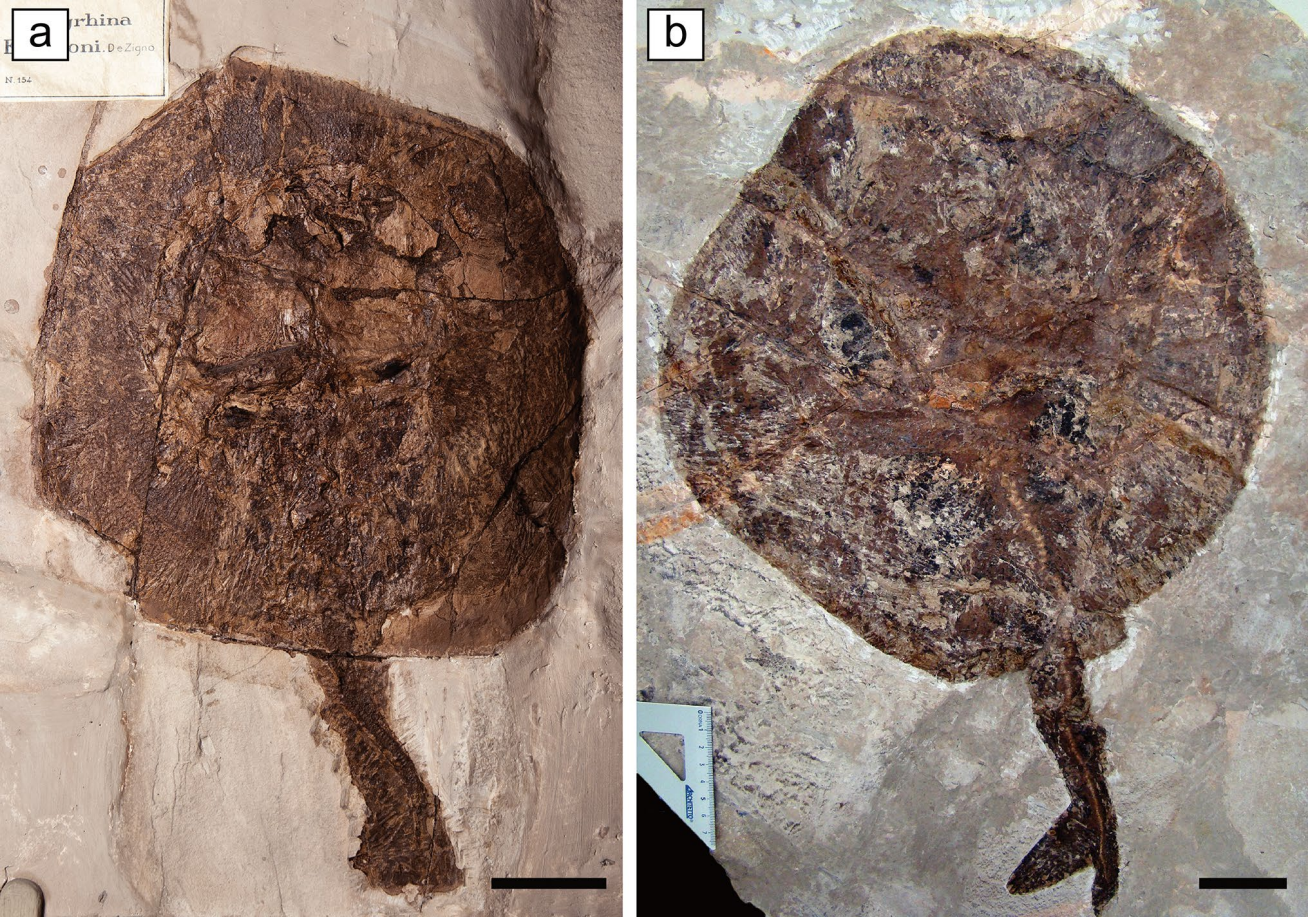
Thornbacks of the family Platyrhinidae from the Eocene Bolca Konservat-Lagerstätte: **a** “*Platyrhina*” *egertoni* (de Zigno, 1876), MGP-PD 154Z, holotype; **b** “*Platyrhina*” sp., MCSNV IG.142530. Scale bars 50 mm

The representatives of the family Platyrhinidae, also known as thornbacks due to the presence of one to three rows of strong thorns on the disk and tail, are also characterised by a notably expanded disk with a broad and rounded snout, not very stout tail, rostral cartilages reduced and not reaching the tip of the snout, pectoral fins narrowly separated anteriorly and extending forward to the end of the snout, two dorsal fins and absence of stings (Compagno 1999; Cappetta 2012). This family is presently represented at Bolca by three species assigned to the genus *Platyrhina* (“*P.*” *gigantea*, “*P.*” *bolcensis*, “*P.*” *egertoni*; see Figs. 12 and 13), although their generic placement appears to be doubtful.

For a long time following their original description in “Ittiolitologia Veronese” (Volta 1796), thornback rays were misidentified as electric rays [e.g., *Raja torpedo* by Volta (1796); *Narcobatus giganteus* by de Blainville (1818); *Narcopterus bolcanus* by Agassiz, (1835); *Torpedo gigantea* by Agassiz (1833–1844) and de Zigno (1874a); *Narcine gigantea* by Molin (1860); *Torpedo egertoni* de Zigno (1876)]. Not until Jaekel (1894) was the Volta holotype (MNHN F.Bol567; Fig. 12a, b) definitively assigned to *Platyrhina*. The species *Narcopterus bolcanus* Agassiz, 1835 also was re-assigned to *Platyrhina* (holotype MGP-PD 8874C/8875C; MGP-PD 26279/80; MGP-PD 26276; Fig. 12c) by Heckel (1851). Lastly, Jaekel (1894) referred another species erected by de Zigno (1876), *Torpedo egertoni* (MGP-PD 154Z; Fig. 13a), to *Platyrhina*. At least two additional specimens from recent excavations tentatively classified as “*Platyrhina*” sp. are housed in MCSNV (Fig. 13b).

Members of the family Platyrhinidae have rarely been recorded in the fossil record, probably because recovered teeth have not been recognized as belonging to this group. A single articulated skeleton from the Late Cretaceous (Turonian) of Morocco referred to as *Tingitanius tenuimandibulus* (Claeson et al. 2013) represents the earliest occurrence of this family in the fossil record. The genus *Britobatos* also was created by Claeson et al. (2013) to include *Raja primarmata* Woodward, 1889 from the Cretaceous of Lebanon, but these authors demonstrated that it represents a stem member of Platyrhinidae, rather than a crown member of this family, as hypothesized by Brito and Dutheril (2004). Additional platyrhinids such as *Cretaplatyrhinoidis* and *Pseudoplatyrhina* are based on isolated teeth from the Late Cretaceous (Santonian) of Europe (Guinot et al. 2012), as well as teeth of *Plathyrhinoidis* and probably *Platyrhina* have been recognized in the Eocene (Lutetian to Priabonian) of Egypt (Underwood et al. 2011). With the exception of *Tingitanius tenuimandibulus* and *Tethybatis selachoides* from the Late Cretaceous of Nardò, southern Italy (de Carvalho 2004), “*Platyrhina*” *gigantea*, “*P*.” *bolcensis* and “*P*.” *egertoni* from the Eocence of Bolca are the only fossil batoids based on articulated skeletal remains that can be confidently assigned to the family Platyrhinidae. Other taxa solely based on isolated teeth such as *Protoplatyrhina renae* from the Late Cretaceous of the USA and the Eocene species “*Platyrhina*” *ypresiensis* from Belgium do not belong to the Platyrhinidae according to de Carvalho (2004) and Cappetta (2012). Although, according to Cappetta (2012), the Bolca material represents “without any doubt” the only fossils that can be assigned to the genus *Platyrhina*. An anatomical and phylogenetic analysis of the material employing robust and up-to-date morphological and phylogenetic methods is needed to definitively confirm this.

Although the exact provenance of the “*Platyrhina*” species from Bolca was not reported in the original descriptions, the preservation quality of the specimens as well as the lithological features of the slabs that include the fossils suggest that this skeletal material most likely derives from the Pesciara site.

### Holocephalii

#### Chimaeriformes

**Fig. 14:**
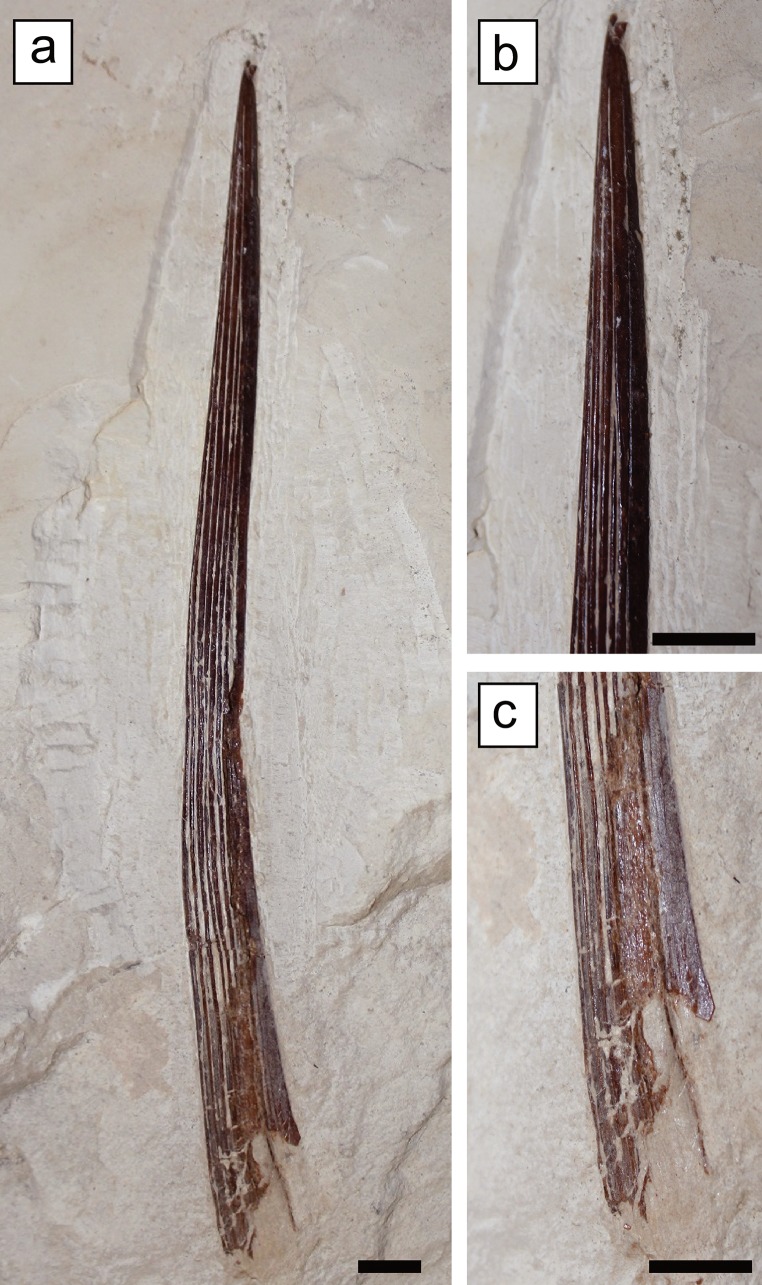
Chimaeroid dorsal-fin spine referable to *Ischyodus* sp. collected during the controlled excavations carried out in the Monte Postale site in 2000: **a** MCSNV IG.VR.61511; **b**, **c** close up of the same specimen. Scale bars 10 mm

Despite the relative abundance of shark and ray specimens in the Eocene Bolca fish assemblage, fossil remains of chimaeroids were unknown from the Bolca deposits up to now. Examination of the chondrichthyan material recovered during the recent 1999–2011 controlled excavations yielded the first chimaeriform specimen that comes from the Monte Postale sites. Chimaeriformes are cartilaginous fishes usually characterised, among other features, by the presence two dorsal fins, the first of which is erectile, with a short base, and preceded by a peculiar diagnostic spine (Stahl 1999). That spine often is the only element preserved in addition to isolated dental plates in the fossil record. An isolated dorsal fin-spine is the single specimen of *Ischyodus* sp. (MCSNV IG.VR.61511; Fig. 14). Although it is still embedded in a limestone slab, such that not all characteristics can be established, it is determined to be laterally compressed and gently curved posteriorly in its basal part, but straight in its upper two thirds. Anteriorly, there seems to be an apico-basal keel. Posterior denticles, which are characteristic for chimaeriform fin-spines, however, are not discernable. The exposed lateral side displays closely arranged and apico-basally extending ridges. The presence of well-developed lateral ridges, the more or less oval cross-section and absence of an anterior concavity readily distinguishes this fin-spine from those of other contemporaneous chimaeriforms such as *Chimaera*, *Callorhynchus* and *Edaphodon* and allows its assignment to the chimeroid *Ischyodus*.

Interestingly, records of Eocene chimaeroids are quite rare and mostly known from their dental plates only. So far, seven species in six genera have been reported from the Ypresian: *Callorhinchus regulbiensis* Gurr, 1962 (England); *Callorhinchus stahli* Kriwet and Gaździcki, 2003 (Antarctica); *Ischyodus dolloi* Leriche, 1902 (England and Antarctica; Ward and Grande 1991); *Edaphodon minor* Ward, 1973 (England); *Amylodon eocenica* (Woodward and White, 1930; England), *Amylodon venablesae* (Casier, 1966; England), *Psaliodus compressus* (Egerton, 1843; England) and *Chimaera eophantasma* Ward, 1973 (England). Additional Eocene chimeroids from either older or younger strata occur, e.g., in the Danian of the USA (*Ischyodus dolloi*, *I*. *williamsae*, *Callorhinchus phillipsi*, *Edaphodon mirificus*, *Elasmodus* sp.; Case 1991, 1996; Cicimurri and Ebersole 2015), Danian of Russia (*Edaphodon eolucifer*; Popov and Yarkov 2001); Thanetian of the USA (*Ischyodus dolloi*, *Callorhinchus alfordi*, *Edaphodon* sp., *Elasmodus hunteri*; Cicimurri and Ebersole 2015), Thanetian of Kazakhstan (*Darbasodus*; Averianov 1991), Thanetian of Belgium (*Edaphodon leptognathus* (Leriche, 1921), Priabonian of Chile (*Ischyodus* sp., *Callorhinchus* sp.; Otero et al. 2013; Otero and Soto-Acuña 2015), early Late Eocene (*Edaphodon* sp.; Parmley and Cicimurri 2005) and also in the Eocene of Germany (*Edaphodon bucklandi*, *Elasmodus hunteri*; Casier 1967; Stahl 1999), Ukraine (*Edaphodon bucklandi*; Stahl 1999), Morocco (probably *Edaphodon bucklandi*; Case and Herman 1973) and Congo (*Paredaphodon arambourgi*; Dartevelle and Casier 1959).

*Ischyodus dolloi* seemingly is one of the chimeroids with the greatest geographic distribution in the Paleogene (Kriwet et al. 2016). Cretaceous records, e.g., from Antarctica are questionable (Hoganson and Erickson 2005). This fossil record account, which is far from being complete, indicates that chimeroids were distributed world-wide but also that our knowledge is strongly biased towards the Northern Hemisphere and especially Europe. The fin-spine of *Ischyodus* sp. reported here from the Ypresian of Bolca represents the first Eocene record of a chimeroid from the Paleogene of Italy and concurrently from southern Europe.

## Discussion

The Eocene ichthyofauna of Bolca is regarded as one of the most diverse fossil fish assemblages of the world, with more than 230 bony and cartilaginous fish species in at least 190 genera from the two main productive sites (Carnevale et al. 2014). The comparison between the Bolca chondrichthyan fauna with those of other almost coeval European Boreal and Mediterranean localities previously reported in the literature (Table 2) provides an almost complete overview of the diversity of the cartilaginous fauna inhabiting the southern Tethys realm and its palaeoenvironmental significance during the early Paleogene. In particular, three consideretions result from these comparisons: (1) Bolca is one of the less diverse Ypresian chondrichthyan assemblages; (2) the Bolca chondrichthyan fauna contains many taxa, most of them small-toothed, which have never been recognized in other tooth-based assemblages; (3) cartilaginous fishes from Bolca are the only ones represented by complete articulated skeletons. In Europe, early to middle Eocene Boreal chondrichthyan faunas have been reported from the London Clay Formation in England (at least 47 taxa; Cooper 1977; Rayner et al. 2009), the Paris basin in France (43 taxa; Dutheil et al. 2006; Adnet and Cappetta 2008), the Lede Sand Formation in Belgium (55 taxa; Nolf 1988; Eeckhaut and De Schutter 2009), the Fürstenau Formation in northern Germany (18 taxa; Diedrich 2012) and Lillebælt Clay in Denmark (31 taxa; Carlsen and Cuny 2014). The tooth-based assemblages from London Clay, Paris basin, Denmark and Belgium are considered as inhabiting cool to temperate, nearshore, high-energy environments with muddy substrate, and are mostly dominated by lamniforms (mainly *Striatolamia macrota* and other odontaspidids), carcharhiniforms and myliobatiforms (see e.g., Cappetta and Ward 1977; Nolf 1988; Eeckhaut and De Schutter 2009; Underwood et al. 2011; Carlsen and Cuny 2014). These assemblages are characterised by a high percentage of demersal/benthic deep-water (or cool shallow) genera including, e.g., *Centrophorus*, *Chlamydoselachus*, *Coupatezia*, *Echinorhinus*, *Heptranchias* and *Pristiophorus*, therefore suggesting that sediments were deposited in rather deep-water contexts, although slight differences in their faunal composition might be driven by other physico-chemical parameters, and food availability (see also Carlsen and Cuny 2014). Cartilaginous fishes from northern Germany probably represent a coastal marine deltaic association, largely influenced by fresh waters (Diedrich 2012). This association is also dominated by *Striatolamia macrota*, but the shark association, although probably shallower than the other Boreal ones, is not considered associatied to tropical or coral reef settings (Diedrich 2012). Moreover, no small teeth have been recognized in this association.

**Table 2:** Occurrences of selachians, batoids and holocephalans in the Early–Middle Eocene of Europe and North Africa

Order	Taxon	Bolca, Italy (a)	London Clay, England (b)	Lede Sand Fm. Belgium (c)	SW France (d)	Paris Basin, France (e)	Trelde Næs, Denmark (f)	North Germany (g)	North Morocco (h)	SW Morocco (i)	Midawara Fm., Egypt (j)
Carcharhiniformes	*Abdounia*		x	x		x			x	x	x
	*Apristurus*				x						
	*Carcharhinus*									x	x
	*Casieria*		x			x					
	*Crassescyliorhinus*										x
	*Eogaleus*	x									
	*Foumtizia*		x	x	x	x	x		x		
	*Galeocerdo*			x				x		x	x
	*Galeorhinus*	x		x		x				x	
	*Gomphogaleus*					x					
	*Hemipristis*									x	x
	*Iago*				x						
	*Leptocharias*										x
	*Megascyliorhinus*		x		x		x				
	*Microscyliorhinus*		x	x		x					
	*Misrichthys*									x	x
	*Mustelus*		x	x		x					
	*Pachygaleus*			x		x		x			
	*Pachyscyllium*			x							
	*Palaeogaleus*								x		
	*Paragaleus*									x	
	*Physogaleus*		x	x	x	x	x		x	x	x
	*Premontreia*			x		x					
	*Rhizoprionodon*					x				x	x
	*Scyliorhinus*		x							x	
	*Sphyrna*									x	
	*Stenoscyllium*								x		
	Triakidae gen. et sp. nov.										x
	*Triakis*		x		x						
	*Trigonotodus*		x								
Chimaeriformes	*Callorhinchus*		x								
	*Chimaera*		x								
	*Edaphodon*			x							
	*Ischyodus*	x	x								
Echinorhiniformes	*Echinorhinus*				x		x		x		
Heterodontiformes	*Heterodontus*		x	x		x					
Hexanchiformes	*Chlamydoselachus*				x		x				
	*Heptranchias*				x		x		x		
	*Hexanchus*		x		x				x		
	*Notorynchus*		x	x			x		x		
	*Weltonia*		x				x		x		
Lamniformes	*Alopias*		x	x	x		x		x	x	x
	*Anomotodon*		x	x	x	x	x	x			
	*Brachycarcharias*	x	x	x		x		x	x		x
	*Carcharias*		x	x		x	x	x	x	x	
	*Carcharocles*		x	x	x		x	x			x
	*Carcharodon*							x			
	*Cretalamna*		x		x		x		x	x	
	*Glueckmanotodus*			x							
	*Hypotodus*		x	x	x	x	x	x	x		
	*Isurolamna*		x	x	x	x	x	x	x		
	*Isurus*							x			
	*Jaekelotodus*		x	x			x	x			
	*Macrorhizodus*		x	x	x		x	x		x	x
	*Mitsukurina*						x				
	*Odontaspis*		x	x	x	x	x	x	x		
	*Otodus*		x	x		x	x	x	x	x	
	*Palaeohypotodus*		x	x		x	x				
	*Parotodus*		x								
	*Striatolamia*		x	x	x	x	x	x	x		
	*Sylvetrilamia*		x	x		x		x			
	*Turania*						x				
	*Usakias*			x			x	x			
	*Woellsteinia*				x		x				
	*Xiphodolamia*		x	x			x	x		x	
Myliobatiformes	*Aetobatus*		x	x					x	x	x
	*Archaeomanta*			x						x	
	*Arechia*					x					
	*Aturobatis*									x	
	*Burnhamia*		x	x		x			x	x	
	*Coupatezia*			x	x	x	x		x		
	*Dasyatis*	x*		x		x				x	x
	*Eomobula*			x		x					
	*Garabatis*									x	
	*Gymnura*			x		x				x	x
	*Heterotorpedo*		x								
	*Himantura*									x	x
	*Hyplolophodon*		x	x		x					
	*Jacquhermania*			x		x					
	*Leidybatis*			x		x					x
	*Lophobatis*					x					
	*Mobula*									x	
	*Myliobatis*		x	x		x			x	x	x
	*Neotrygon*										x
	*Ouledia*					x				x	x
	*Promyliobatis*	x									
	*Pseudaetobatus*								x		
	*Rhinoptera*									x	
	*Taeniura*										x
	*Urolophus*	x*									
Orectolobiiformes	*Chiloscyllium*					x				x	
	*Eostegostoma*			x	x						x
	*Ginglymostoma*					x			x		
	*Hemiscyllium*			x	x						x
	*Nebrius*			x		x				x	x
	*Palaeorhincodon*			x		x					
	*Pararhincodon*		x		x						x
	*Protoginglymostoma*			x							
Pristiformes	*Anoxypristis*									x	x
	*Pristis*		x	x		x				x	x
	*Propristis*									x	x
Pristiophoriformes	*Pristiophorus*				x		x				
Rajiformes	*Platyrhina*	x*									x
	*Platyrhinoidis*										x
	*Raja*		x								
	*Rhinobatos*	x*	x	x	x	x			x	x	
	*Rhynchobatos*			x		x				x	x
	*Smithraja*			x	x						
Selachiii inc. sedis	*Odontorhytis*									x	
Squaliformes	*Centrophorus*				x		x				
	*Isistius*		x	x	x		x		x		
	*Megasqualus*		x	x							
	*Squalus*		x			x					
Squatiniformes	*Squatina*		x	x	x	x	x		x		
Torpediniformes	*Eotorpedo*			x							
	*Narcine*				x					x	x
	*Titanonarke*	x									
	*Torpedo*				x						

Data from: (a) Carnevale et al. (2014), Marramà et al. (2017a, b); (b) Cooper (1977), Rayner et al. (2009); (c) Nolf (1988), Eeckhaut and De Schutter (2009); (d) Adnet (2006), Adnet et al. (2008); (e) Dutheil et al. (2006), Adnet and Cappetta (2008); (f) Carlsen and Cuny (2014); (g) Diedrich (2012); (h) Noubhani and Cappetta (1997); (i) Adnet et al. (2010); (j) Underwood et al. (2011)

*The asterisks indicate that revision of these taxa from Bolca will most likely reveal the existence of remarkable morphological differences between these fossils and extant taxa, resulting in separate generic placements

Almost coeval Eocene chondrichthyan assemblages are reported in the Mediterranean (Tethyan) area and are known from southwestern France (31 taxa; Adnet 2006; Adnet et al. 2008), Northern Morocco (25 taxa; Noubhani and Cappetta 1997), the Ad-Dakhla region of southwestern Morocco (38 taxa; Adnet et al. 2010) and the Midawara Formation of the Fayum area in Egypt (34 taxa; Underwood et al. 2011). From a palaeoenvironmental point of view, deep-water genera from southwestern France and North Morocco such as *Chlamidoselachus*, *Heptranchias*, *Hexancus*, *Centrophorus*, *Echinorhinus*, *Coupatezia* and *Pristiophorus* suggest deposition in cool, deep water (Noubhani and Cappetta 1997; Adnet 2006; Adnet et al. 2008; Carlsen and Cuny 2014). On the contrary, the assemblage of southwestern Morocco (38 taxa) is regarded as a tropical shallow marine deposit in proximity to an emerged coastal area for the presence of several demersal taxa belonging to carcharhinids, pristids and myliobatiforms (Adnet et al. 2010). The authors considered this association similar to that of the Middle Eocene Midawara Fm. of the Fayum area in Egypt, considered by Underwood et al. (2011) typical of open shelf, relatively shallow deposits, rich in invertebrate and fish faunas. The latter, in particular, is dominated by small shallow-water predatory sharks, as the carcharinids *Galeocerdo* and *Carcharhinus* (the latter only present in Fayum area and SW Morocco) and triakids, all considered generalist feeders preying on small active prey (Underwood et al. 2011). Cosmopolitan specialized feeders on small nectobenthic prey are also represented in the Midawara Fm. by the small odontaspidid *Brachycarcharias,* pristids, rhinobatids and some myliobatiforms (e.g., *Gymnura*; Underwood et al. 2011).

There is little taxonomic overlap among the Bolca sites with the almost coeval boreal assemblages of northern and southwestern France, Belgium and England, and the Tethyan North Marocco: 3 of at least 17 identified species in 10 genera (*Galeorhinus*, *Ischyodus* and *Brachycarcharias*). Although isolated teeth possibly referred to the batoids *Dasyatis* and *Rhinobatos* were found in Eocene assemblages of Belgium, France, England and northern Morocco, the presence of these taxa in the Bolca ichthyofauna is only tentative. A revision of the Bolca “*Dasyatis*” and “*Rhinobatus*” species will most likely reveal the existence of considerable differences between these fossil and extant taxa, resulting in separate generic placements. Only the cosmopolitan *Brachycarcharias* is shared with the German assemblage, whereas there are not common taxa with the Denmark association. The different taxonomic composition of the Bolca and other Eocene European chondrichthyan assemblages appear to be largely related to different palaeoenvironmental conditions, and faunal differences are, therefore, interpreted as purely ecological. Several studies evidenced the intimate relationships between the Eocene ichthyofauna of Bolca and a variety of shallow-water biotopes, including those characterised by the presence of coral reefs (Bellwood 1996; Landini and Sorbini 1996). The Eocene ichthyofauna of Bolca includes the earliest occurrences of many acanthomorph lineages in the fossil record, and it is traditionally regarded as the earliest coral reef fish assemblage of modern type (Patterson 1993; Bellwood 1996; Marramà et al. 2016b, c). A recent quantitative palaeoecological and taphonomic analysis of the fish assemblages of the two main sites of Bolca suggested that the fossiliferous sediments of the Monte Postale site were deposited close to an emerged coastal area characterised by mangroves and seagrass, in a coral reef context (Marramà et al. 2016a; see also Vescogni et al. 2016). Sedimentological and taphonomic features suggest that the sediments of the Pesciara site were deposited in a intraplatform basin in which anoxic conditions at the bottom and biofilms acted as promoters of high-quality fossil preservation, apparently without a direct influence of coral reefs (Papazzoni and Trevisani 2006; Marramà et al. 2016a). In this perspective, the Bolca palaeoenvironmental and palaeoecological characters appear to be more consistent with the tropical shallow settings reported from southwestern Morocco and, even more, with those of the Fayum area in Egypt. Like the latter, in particular, the Bolca fauna is characterized by the presence of small odontaspidids (*Brachycarcharias*), small carcharhinids (*Eogaleus*) and juvenile triakids (*Galeorhinus*), all generalist feeders on small nectobenthic prey and zooplanktivorous coastal bony fishes such as clupeoids, which represented one of the main trophic resources in the Bolca palaeobiotope (Marramà et al. 2016a). As a remarkable note, the presence of thornbacks of the family Platyrhinidae, represented in Bolca by at least three species (“*Platyrhina*” *bolcensis*, “*P*.” *gigantea* and “*P*.” *egertoni*), was only reported from the Fayum area (Egypt) with the genera *Platyrhina* and *Platyrhinoidis*, therefore suggesting closer palaeoenvironmental features between these two deposits than the others. Differences in taxonomic composition appear to be related to the presence of a coral reef setting detected for the Bolca associations but not for the other coeval boreal or tethyan assemblages. In fact, the chondrichthyan assemblage structure of Bolca seems to be consistent with its palaeoenvironmental interpretations. The extant *Galeorhinus* inhabits warm temperate and tropical waters on continental shelves and juvenile individuals can be relatively common in reef contexts (Compagno 2003). The extinct genus *Eogaleus* was apparently also reported in lower Eocene shallow water contexts of the Cambay Shale beds of India (Rana et al. 2004). The presence of isolated teeth of the cosmopolitan odontaspidid genus *Brachycarcharias*, representing an opportunistic top predator of more open water contexts (e.g., Cappetta and Nolf 2005; Underwood et al. 2011) may suggest sporadic incursions of this predator into the Bolca shallow water palaeobiotopes (Marramà et al. 2017b). Modern electric rays of the family Narcinidae (numbfishes) are a group of tropical inshore to deep-water (up to about 1000 m of depth, but usually below 250 m) batoids, mostly occurring off soft sandy beaches and in muddy enclosed bays, often associated with coral reefs (Carvalho et al. 1999; McEachran and Carvalho 2002). Recent rhinobatids and platyrhinids are mostly found in warm-temperate to tropical inshore continental waters, mostly occurring off sandy beaches, in muddy enclosed bays, near kelp beds and shallow mud bottom (Compagno and Last 1999a, b). Urolophids are temperate to tropical inshore to deep-water batoids often ranging from the intertidal to the upper slope on soft bottoms down to 420 m of depth (Last and Compagno 1999a). Modern dasyatids are mostly demersal inshore batoids, although some species occur offshore onto the continental margins or along the upper continental slopes (Last and Compagno 1999b). Extant myliobatids range from the intertidal to the upper slope on soft and hard bottom, mostly occurring around coral and rocky reefs, kelp beds, lagoons and enclosed and open bays (Compagno and Last 1999c). Finally, although living chimaeroids mostly inhabit deep waters, some species are known to venture into shallow areas to feed or to breed (Bigelow and Schroeder 1953), and fossils remains have been found in shallow water contexts (see e.g., Kriwet and Gaździcki 2003; Takeuchi and Huddleston 2006; Kriwet and Klug 2011).

In this perspective, the presence of shallow water selachians (carcharhiniforms), batoids (rajiforms, myliobatiforms, torpediniforms) and holocephalans (chimaeriformes) in the Eocene ichthyofauna of the Bolca Konservat-Lagerstätte is in good accordance with the inferred shallow water habitats associated with reefs for the Bolca sites. Moreover, it is likely that the Bolca setting represented a unique and distinct refuge area for this ichthyofauna in the entire Tethys Sea at least during the Early–Middle Eocene.

As already highlighted above, unlike the Bolca deposits where chondrichthyans are mainly represented by complete and articulated skeletons, the other Boreal and Mediterranean assemblages are solely known from their isolated teeth. This can be explained, at least in part, by the type of depositional context and taphonomic conditions, since the Bolca deposit is the only one that can be considered as a Konservat-Lagerstätte, whereas the other ones are clearly of Konzentrat-type. The extraordinary diversity of the teleost fauna of the Bolca Lagerstätte is in strong contrast with the reduced number of chondrichthyan taxa (having the lowest number of genera among all Boreal and Tethyan Eocene assemblages considered in this study). We hypothesize that the low diversity of cartilaginous fishes in Bolca may reflect a real biological and ecological signal, rather than the product of collection and/or taphonomic biases based on the high-quality preservation of the fossils recovered so far.

## Conclusions

Although the Eocene chondrichthyans from Bolca are mentioned in literature at least since the end of the eighteenth century (Volta 1796), the systematic position and relationships of most selachians and batoids have not been tested with modern comprehensive cladistics analyses. A phylogenetic interpretation of the Eocene ichthyodiversity would represent a key tool to understand diversification patterns of chondrichthyan fishes after the K–Pg boundary in a palaeobiogeographic and palaeoclimatological context. The results deriving from the revision of the chondrichthyan material from Bolca could be compared and integrated with those already detected for the actinopterygians in the context of the Early Paleogene fish radiation (Guinot and Cavin 2016; Marramà et al. 2016b, c). New detailed studies on the chondrichthyans of Bolca will largely contribute not only to fill the gaps in our understanding of the evolutionary history of cartilaginous fishes, but also to improve the knowledge of one of the most important palaeontological sites in the world and, consequently, to the palaeontological heritage of Europe.

## References

[CR1] Adnet C (2006). Nouvelles faunes de selaciens (Elasmobranchii, Neoselachii) de l’Eocene moyen des Landes (Sud–Ouest, France). Implication dans la connaissance des communautes d’eaux profondes. Palaeo Ichthyologica.

[CR2] Adnet S, Cappetta H (2008). New fossil triakid sharks from the early Eocene of Prémontré, France, and comments on fossil record of the family. Acta Palaeontologica Polonica.

[CR3] Adnet S, Cappetta H, Reynders J (2008). Contribution of Eocene sharks and rays from Southern France to the history of deep-sea selachians. Acta Geologica Polonica.

[CR4] Adnet S, Cappetta H, Tabuce R (2010). A Middle-Late Eocene vertebrate fauna (marine fish and mammals) from southwestern Morocco; preliminary report: age and palaeobiogeographical implications. Geological Magazine.

[CR5] Agassiz, L. 1833–1844. *Recherches sur les Poissons fossiles*. Neuchâtel: Petitpierre.

[CR6] Agassiz L (1835). Revue critique des Poissons fossiles figurés dans l’Ittiolitologia Veronese.

[CR7] Applegate SP (1978). Phyletic studies. Part. I. Tiger sharks. Universidad Nacional Autonoma de Mexico, Instituto de Geologia Revista.

[CR8] Arambourg C (1952). Les vertébrés fossiles des gisements de phosphates (Maroc-Algeérie-Tunisie). Notes et Mémoires du Service Géologique du Maroc.

[CR9] Aschliman NC, Claeson KM, McEachran JD, Carrier JC, Musick JA, Heithaus MR (2012). Phylogeny of Batoidea. Biology of Sharks and Their Relatives.

[CR10] Aschliman NC, Nishida M, Miya M, Inoue JG, Rosana KM, Naylor GJP (2012). Body plan convergence in the evolution of skates and rays (Chondrichthyes: Batoidea). Molecular Phylogenetics and Evolution.

[CR11] Averianov AO (1991). A new genus of Paleocene chimaeroid fishes from Kazakhstan. Paleontologicheskiy zhurnal.

[CR12] Bannikov AF (2004). Fishes from the Eocene of Bolca, northern Italy, previously classified with the Chaetodontidae (Perciformes). Studi e Ricerche sui Giacimenti Terziari di Bolca.

[CR13] Bannikov AF (2006). Fishes from the Eocene of Bolca, northern Italy, previously classified in the Sparidae, Serranidae and Haemulidae (Perciformes). Geodiversitas.

[CR14] Bannikov AF (2008). Revision of the atheriniform fish genera *Rhamphognathus* Agassiz and *Mesogaster* Agassiz (Teleostei) from the Eocene of Bolca, northern Italy. Studi e Ricerche sui Giacimenti Terziari di Bolca.

[CR15] Bassani F (1897). Aggiunte all’ittiofauna Eocenica dei Monti Bolca e Postale. Palaeontographia Italica.

[CR16] Bellwood DR (1996). The Eocene fishes of Monte Bolca: the earliest coral reef fish assemblage. Coral Reefs.

[CR17] Bigelow H, Schroeder WC (1953). Fishes of the Western Atlantic. Part Two: Sawfishes, Guitarfishes, Skates and Rays. Chimaeroids. Memoir of the Sears Foundation for Marine Research.

[CR18] de Blainville HD (1818). Sur les ichthyolites ou les poissons fossiles. Nouveau Dictionnaire d’Histoire Naturelle.

[CR19] Blot J (1969). Les poissons fossiles du Monte Bolca classés jusqu’ici dans les familles des Carangidae, Menidae, Ephippidae, Scatophagidae. Studi e Ricerche sui Giacimenti Terziari di Bolca.

[CR20] Blot J (1980). La faune ichthyologique des gisements du Monte Bolca (Province de Verone, Italie). Catalogue systématique présentant l’état actuel des 160 recherches concernant cette faune. Bulletin du Muséum national d’histoire naturelle Paris.

[CR21] Brito PM, Dutheil DB, Arratia G, Tintori A (2004). A preliminary systematic analysis of Cretaceous guitarfishes from Lebanon. Mesozoic fishes 3.

[CR22] Cappetta H (1975). Les Sélaciens éocènes du Monte-Bolca. I—Les Carcharhinidae. Studi e Ricerche sui Giacimenti Terziari di Bolca.

[CR23] Cappetta H (1980). Les selaciens du Cretace superieur du Liban. I: Requins. Palaeontographica A.

[CR24] Cappetta H (1980). Modification du statut générique de quelque espèces de sélaciens crétacés et tertiaires. Palaeovertebrata.

[CR25] Cappetta H (1987). Handbook of Paleoichthyology - Chondrichthyes II-Mesozoic and Cenozoic Elasmobranchii.

[CR26] Cappetta H (1988). Les Torpediniformes (Neoselachii, Batomorphii) des phosphates du Maroc. Observations sur la denture des genres actuels. Tertiary Research.

[CR27] Cappetta H (2012). Handbook of Paleoichthyology—Chondrichthyes—Mesozoic and Cenozoic Elasmobranchii: Teeth.

[CR28] Cappetta H, Nolf D (2005). Revision of some Odontaspididae (Neoselachii: Lamniformes) from the Paleocene and Eocene of the North Sea Basin. Bulletin de l’Institut Royal des Sciences Naturelles de Belgique.

[CR29] Cappetta H, Pfeil F, Schmidt-Kittler N (2000). New biostratigraphical data on the marine Upper Cretaceous and Palaeogene of Jordan. Newsletters on Stratigraphy.

[CR30] Cappetta H, Ward DJ (1977). A new Eocene shark from the London Clay of Essex. Palaeontology.

[CR31] Carlsen AW, Cuny G (2014). A study of the sharks and rays from the Lillebælt Clay (Early–Middle Eocene) of Denmark, and their palaeoecology. Bulletin of the Geological Society of Denmark.

[CR32] Carnevale G, Bannikov AF, Marramà G, Tyler JC, Zorzin R, Papazzoni CA, Giusberti L, Carnevale G, Roghi G, Bassi D, Zorzin R (2014). The Pesciara-Monte Postale Fossil-Lagerstätte: 2. Fishes and other vertebrates. The Bolca Fossil-Lagerstätte: A window into the Eocene World.

[CR33] Carnevale G, Pietsch TW (2009). An Eocene frogfish from Monte Bolca, Italy: the earliest skeletal record for the family. Palaeontology.

[CR34] Carnevale G, Pietsch TW (2010). Eocene handfishes from Monte Bolca, with description of a new genus and species, and a phylogeny of the family Brachionichthyidae (Teleostei: Lophiiformes). Zoological Journal of the Linnean Society.

[CR35] Carnevale G, Pietsch TW (2011). Batfishes from the Eocene of Bolca. Geological Magazine.

[CR36] Carnevale G, Pietsch TW (2012). †*Caruso*, a new genus of anglerfishes from the Eocene of Bolca, Italy, with a comparative osteology and phylogeny of the teleost family Lophiidae. Journal of Systematic Palaeontology.

[CR153] Carnevale, G., Johnson, G.D., Marramà, G., and A.F. Bannikov. 2017. A reappraisal of the Eocene priacanthid fish *Pristigenys substriata* (De Blainville, 1818) from Monte Bolca, Italy. *Journal of Paleontology* 91 (3): 554–565.

[CR37] de Carvalho MR, Arratia G, Tintori A (2004). A Late Cretaceous thornback ray from southern Italy, with a phylogenetic reappraisal of the Platyrhinidae (Chondrichthyes: Batoidea). Mesozoic fishes 3—systematics, paleoenvironments and biodiversity.

[CR38] de Carvalho MR, Elliott DK, Maisey JG, Yu X, Miao D (2010). Morphology and phylogenetic relationships of the giant electric ray from the Eocene of Monte Bolca, Italy (Chondrichthyes: Torpediniformes). Morphology, phylogeny and paleobiogeography of fossil fishes.

[CR39] de Carvalho MR, Compagno LJV, Lat PR, Carpenter KE, Niem VH (1999). Torpediniformes: Narcinidae, Numbfishes. FAO species identification guide for fishery purposes. The living marine resources of theWestern Central Pacific. Batoid fishes, chimaeras and bony fishes part 1 (Elopidae to Linophrynidae),.

[CR40] de Carvalho MR, Maisey JC, Grande L (2004). Freshwater stingrays of the Green River formation of Wyoming (Early Eocene), with the description of a new genus and species and an analysis of its phylogenetic relationships (Chondrichthyes: Myliobatiformes). Bulletin of the American Museum of Natural History.

[CR41] Case GR (1991). A new species of chimaeroid fish from the Upper Paleocene (Thanetian) of Maryland, USA. Palaeovertebrata.

[CR42] Case GR (1996). A new selachian fauna from the Lower Hornerstown Formation (Early Paleocene/Montian) of Monmouth County, New Jersey. Palaeontographica A.

[CR43] Case GR, Herman J (1973). A dorsal fin spine of the chimaeroid fish, *Edaphodon* cf. *bucklandi* (Agassiz) from the Eocene of Morocco. Bulletin de la Société Belge de Géologié, de Paléontologie et d’Hydrologie.

[CR44] Case GR, Cook TD, Wilson MVH (2015). A new elasmobranch assemblage from the early Eocene (Ypresian) Fishburne Formation of Berkeley County, South Carolina, USA. Canadian Journal of Earth Sciences.

[CR45] Casier E (1946). La faune ichthyologique de l’Yprésien de la Belgique. Mémoires du Musée Royal d’Histoire Naturelle de Belgique.

[CR46] Casier E (1967). Le Landénien de Dormaal (Brabant) et sa faune ichthyologique. Institut Royal des Sciences Naturelles de Belgique, Mémoires.

[CR47] Castro JI (1993). The shark nursery of Bulls Bay, South Carolina, with a review of the shark nurseries of the southeastern coast of the United States. Environmental Biology of Fishes.

[CR48] Cicimurri DJ, Ebersole JA (2015). Paleocene chimaeroid fishes (Chondrichthyes: Holocephali) from the eastern United States, including two new species of *Callorhinchus*. PaleoBios.

[CR49] Claeson KM (2014). The impacts of comparative anatomy of electric rays (Batoidea: Torpediniformes) on their systematic hypotheses. Journal of Morphology.

[CR50] Claeson KM, Underwood CJ, Ward DJ (2013). †*Tingitanius tenuimandibulus*, a new platyrhinid batoid from the Turonian (Cretaceous) of Morocco and the Cretaceous radiation of the Platyrhinidae. Journal of Vertebrate Paleontology.

[CR51] Compagno LJV, Hamlett WC, Niem VH (1999). Chapter 1. Systematics and body form. Sharks, skates and rays. The Biology of Elasmobranch Fishes.

[CR53] Compagno LJV, Last PR, Carpenter KE, Niem VH (1999). Rhinobatidae. FAO species identification guide for fishery purposes. The living marine resources of theWestern Central Pacific. Batoid fishes, chimaeras and bony fishes part 1 (Elopidae to Linophrynidae).

[CR54] Compagno LJV, Last PR, Carpenter KE, Niem VH (1999). Platyrhinidae. FAO species identification guide for fishery purposes. The living marine resources of theWestern Central Pacific. Batoid fishes, chimaeras and bony fishes part 1 (Elopidae to Linophrynidae).

[CR55] Carvalho LJV, Last PR, Carpenter KE, Niem VH (1999). Myliobatidae. In FAO species identification guide for fishery purposes. The living marine resources of theWestern Central Pacific. Batoid fishes, chimaeras and bony fishes part 1 (Elopidae to Linophrynidae).

[CR52] Compagno LJV (2003). Sharks of the order Carcharhiniformes.

[CR56] Cooper J (1977). The palaeontology of the London Clay (Lower Eocene) of the Herne Bay coastal section, Kent. England. Proceedings of the Geological Association.

[CR57] Culver SJ, Rawson PF (2000). Biotic response to global change. The last 145 million years.

[CR157] Cuvier, G.L.C.F.D. 1816.* Le Règne Animal distribué d'après son organisation pour servir de base à l'histoire naturelle des animaux et d'introduction à l'anatomie comparée. Les reptiles, les poissons, les mollusques et les annélides.* Paris: Deterville.

[CR58] D’Erasmo G (1922). Catalogo dei pesci fossili delle Tre Venezie. Memorie dell’Istituto di Geologia della Regia Università di Padova.

[CR59] Dartevelle E, Casier E (1959). Les poissons fossiles du Bas-Congo et des régions voisines (troisième partie). Annales du Musée Royal du Congo Belge, Tervuren, A (série III).

[CR60] Diedrich, C.G. 2012. Eocene (Lutetian) Shark-rich coastal paleoenvironments of the Southern North Sea Basin in Europe: Biodiversity of the marine Fürstenau Formation Including Early white and megatooth sharks. *International Journal of Oceanography*, p 565326. doi:10.1155/2012/565326.

[CR61] Dutheil DB, Moreau F, De Plöeg G (2006). Les ichthyofaunes du gisement à ambre de Le Quesnoy (Paléocène et Éocène du bassin de Paris, France). Cossmanniana.

[CR62] Eastman CR (1904). Description of Bolca fishes. Bulletin of the Museum of Comparative Zoology.

[CR63] Eastman CR (1905). Les types de Poissons fossiles du Monte Bolca au Muséum d’Histoire Naturelle de Paris. Mémoires de la Société géologique de France.

[CR64] Eeckhaut CR (1911). Catalog of the fossil fishes in the Carnegie Museum. Part H. Supplement to the catalog of fishes from the Upper Eocene of Monte Bolca. Memoirs of the Carnegie Museum.

[CR65] Eeckhaut G, De Schutter P (2009). The elasmobranch fauna of the Lede Sand Formation at Oosterzele (Lutetian, Middle Eocene of Belgium). Palaeofocus.

[CR66] Fanti F (1914). La serie stratigrafica del Monte Bolca e dei suoi dintorni. Memorie dell‟Istituto di Geologia della Regia Università di Padova.

[CR67] Fanti F (1915). Il Paleogene del Veneto. Memorie dell‟Istituto di Geologia della Regia Università di Padova.

[CR68] Fanti F, Minelli D, Larocca Conte G, Miyashita T (2016). An exceptionally preserved Eocene shark and the rise of modern predatory-prey interaction in the coral reef food web. Zoological Letters.

[CR69] Fischer J, Voigt S, Schneider JW, Buchwitz M, Voigt S (2011). A selachian freshwater fauna from the Triassic of Kyrgyzstan and its implication for Mesozoic shark nurseries. Journal of Vertebrate Paleontology.

[CR70] Frigo M, Sorbini L (1997). 600 fossili per Napoleone: catalogo della mostra.

[CR71] Gaudant J (1997). La querelle des trois abbés (1793–1795): Le débat entre Domenico Testa, Alberto Fortis et Giovanni Serafino Volta sur la signification des poissons pétrifiés du Monte Bolca (Italie). Studi e Ricerche sui Giacimenti Terziari di Bolca.

[CR72] Gaudant J (2011). Brève histoire de la collection Gazola de poissons fossiles éocène du Monte Bolca (Italie) conservée au Muséum National d’Histoire naturelle, Paris. Geodiversitas.

[CR73] Gingerich PD (2006). Environment and evolution through the Paleocene-Eocene thermal maximum. Trends in Ecology & Evolution.

[CR155] Gorjanovic-Kramberger, D. 1885. Palaeoichthyolozki prilozi. *Rad jugoslavenska Akademije Znanosti i Umjetnosti* 72 (4):10–65.

[CR74] Guerra R, Zorzin R (2014). Bibliografia e citazioni di Bolca. Opere dal 1550 al 1850 (primo contributo). Studi e ricerche sui giacimenti terziari di Bolca, XV – Miscellanea paleontologica.

[CR75] Guinot G, Cappetta H, Underwood CJ, Ward DJ (2012). Batoids (Elasmobranchii: Batomorphii) from the British and French Late Cretaceous. Journal of Systematic Palaeontology.

[CR76] Guinot G, Cavin L (2016). ‘Fish’ (Actinopterygii and Elasmobranchii) diversification patterns through deep time. Biological Reviews.

[CR159] Gurr, P.R. 1962. A new fishfauna from the Woolwich Bottom Beds (Sparnacian) of Herne Bay, Kent.* Proceedings of the Geologists' Association* 73(4): 419–447.

[CR77] Heckel MJ (1851). Bericht einer auf Kosten der kais. Akademie der Wissenschaften durch Oberösterreich nach Salzburg, München, Innsbruck, Bozen, Verona, Padua, Venedig und Triest unternommenen Reise. Sitzungsberichte der Kaiserlichen Akademie der Wissenschaften (Mathematisch-naturwissenschaftliche Klasse).

[CR78] Heckel MJ (1853). Bericht über die vom Herrn Cavalière Achille de Zigno hier angelangte Sammlung fossiler Fische. Sitzungsberichte der Kaiserlichen Akademie der Wissenschaften. Mathematisch-Naturwissenschaftliche Klasse.

[CR79] Heupel MR, Carlson JK, Simpfendorfer CA (2007). Shark nursery areas: concepts, definition, characterization and assumptions. Marine Ecology Progress Series.

[CR80] Hoganson JW, Erickson JM (2005). A new species of *Ischyodus* (Chondrichthyes: Holocephali: Callorhynchidae) from Upper Maastrichtian shallow marine facies of the Fox Hills and Hell Creek Formations, Williston Basin, North Dakota. USA. Palaeontology.

[CR81] Hovestadt DC, Hovestadt-Euler M, Micklich N (2010). A review of the chondrichthyan fauna of Grube Unterfeld (Frauenweiler) clay pit. Kaupia.

[CR82] Jaekel O (1894). Die eocänen Selachier vom Monte Bolca: ein Beitrag zur Morphogenie der Wirbelthiere.

[CR84] Kriwet J, Engelbrecht A, Mörs T, Reguero M, Pfaff C (2016). Ultimate Eocene (Priabonian) chondrichthyans (Holocephali, Elasmobranchii) of Antarctica. Journal of Vertebrate Paleontology.

[CR85] Kriwet J, Gaździcki A (2003). New Eocene Antarctic chimeroid fish (Holocephali, Chimaeriformes). Polish Polar Research.

[CR86] Kriwet J, Klug S (2011). An embryonic mandibular tooth plate and associated remains of a Late Jurassic chimaeroid (Holocephali, Chimaeriformes) from the Iberian Peninsula. Journal of Vertebrate Paleontology.

[CR87] Kriwet J, Soler-Gijón R, López-Martínez N (2007). Neoselachians from the Upper Campanian and Lower Maastrichtian (Upper Cretaceous) of the Southern Pyrenees, Northern Spain. Palaeontology.

[CR88] Landini W, Sorbini L, Cherchi A (1996). Ecological and trophic relationships of Eocene Monte Bolca (Pesciara) fish fauna. Autecology of selected fossil organisms: achievements and problem.

[CR89] Last PR, Compagno LJV, Carpenter KE, Niem VH (1999). Urolophidae. FAO species identification guide for fishery purposes. The living marine resources of theWestern Central Pacific, batoid fishes, chimaeras and bony fishes part 1 (Elopidae to Linophrynidae),.

[CR90] Last PR, Compagno LJV, Carpenter KE, Niem VH (1999). Dasyatidae. FAO species identification guide for fishery purposes. The living marine resources of theWestern Central Pacific, batoid fishes, chimaeras and bony fishes part 1 (Elopidae to Linophrynidae).

[CR91] Lear CH, Bailey TR, Pearson PN, Coxall HK, Rosenthal Y (2008). Cooling and ice growth across the Eocene-Oligocene transition. Geology.

[CR160] Leriche, M. 1902. Les poissons paléocenes de la Belgique. *Mémoires du Musée Royal d’Histoire Naturelle de Belgique* 2: 1–48.

[CR161] Leriche, M. 1921. Sur les restes de Poissons remaniés dans le Néogène de la Belgique. Leur signification au point de vue de l'histoire géologique de la Belgique pendant le tertiaire supérieur. *Bulletin de la Société Belge de Géologié* 30: 115–120

[CR93] Lioy P (1865). Sopra alcuni avanzi di plagiostomi fossili del Vincentino e specialmente sull’ *Alopiopsis plejodon* Lioy (*Galeus cuvieri* Ag.). Atti della Società Italiana di Scienze Naturali e del Museo Civico di Storia Naturale di Milano.

[CR94] Marramà G, Bannikov AF, Tyler JC, Zorzin R, Carnevale G (2016). Controlled excavations in the Pesciara and Monte Postale sites provide new insights about the paleoecology and taphonomy of the fish assemblages of the Eocene Bolca Konservat-Lagerstätte, Italy. Palaeogeography, Palaeoclimatology, Palaeoecology.

[CR95] Marramà G, Carnevale G (2015). Eocene round herring from Monte Bolca, Italy. Acta Palaeontologica Polonica.

[CR96] Marramà G, Carnevale G (2015). The Eocene sardine †*Bolcaichthys catopygopterus* (Woodward, 1901) from Monte Bolca, Italy: osteology, taxonomy and paleobiology. Journal of Vertebrate Paleontology.

[CR97] Marramà G, Carnevale G (2016). An Eocene anchovy from Monte Bolca, Italy: The earliest known record for the family Engraulidae. Geological Magazine.

[CR98] Marramà, G., and G. Carnevale. 2017a. Morphology, relationships and paleobiology of the Eocene barracudina *Holosteus esocinus* (Aulopiformes, Paralepididae) from Monte Bolca, Italy. *Zoological Journal of the Linnean Society* 181:209–228.

[CR151] Marramà, G., and G. Carnevale. 2017b. *Eoalosa janvieri *gen. et sp. nov., a new clupeid fish (Teleostei, Clupeiformes) from the Eocene of Monte Bolca, Italy. *PalZ. Paläontologische Zeitschrift*. doi:10.1007/s12542-017-0378-0 10.1007/s12542-017-0378-0 PMC5830460 29515269

[CR152] Marramà, G., Claeson, K.M, Carnevale, G., and J. Kriwet. 2017a. Revision of Eocene electric rays (Torpediniformes, Batomorphii) from the Bolca Konservat-Lagerstätte, Italy, reveals the first fossil embryo in situ in marine batoids and provides new insights into the origin of trophic novelties in coral reef fishes. *Journal of Systematic Palaeontology. *doi:10.1080/14772019.2017.1371257 10.1080/14772019.2017.1371257 PMC6130837 30210265

[CR99] Marramà G, Engelbrecht A, Carnevale G, Kriwet J (2017). Eocene sand tiger sharks (Lamniformes, Odontaspididae) from the Bolca Konservat-Lagerstätte, Italy: Palaeobiology, palaeobiogeography and evolutionary significance. Historical Biology.

[CR100] Marramà G, Garbelli C, Carnevale G (2016). A morphospace for the Eocene fish assemblage of Bolca, Italy: A window into the diversification and ecological rise to dominance of modern tropical marine fishes. Bollettino della Società Paleontologica Italiana.

[CR101] Marramà G, Garbelli C, Carnevale G (2016). A clade-level morphospace for the Eocene fishes of Bolca: Patterns and relationships with modern tropical shallow marine assemblages. Bollettino della Società Paleontologica Italiana.

[CR102] Mattioli PA (1550). Petri Andreae Matthioli Medici Senensis Commentarii, in Libros sex Pedacii Dioscoridis Anazarbei, de Materia Medica, Adjectis quàm plurimis plantarum & animalium imaginibus, eodem authore, detti Commentarii.

[CR103] McEachran JD, Aschliman N, Carrier JC, Musick JA, Heithaus MR (2004). Phylogeny of Batoidea. Biology of sharks and their relatives.

[CR104] McEachran JD, de Carvalho MR, Miyake T, Carpenter K (2002). Batoid fishes. The living marine resources of the Western Central Atlantic. FAO species identification guide for fishery purposes and American Society of Ichthyologists and Herpetologists 5.

[CR105] McEachran JD, Dunn KA, Miyake T, Stassney MLJ, Parenti LR, Johnson GD (1996). Interrelationships of the batoid fishes (Chondrichthyes: Batoidea). Interrelationships of fishes.

[CR106] Molin R (1860). Primitiae Musei Archigymnasii patavini. Sitzungsberichte der Kaiserlichen Akademie der Wissenschaften. Mathematisch-Naturwissenschaftliche Klasse.

[CR107] Molin R (1861). De Rajidis tribus bolcanis. Sitzungsberichte der Kaiserlichen Akademie der Wissenschaften (Mathematisch-naturwissenschaftliche Klasse).

[CR108] Nelson JS (2006). Fishes of the world.

[CR109] Nelson JS, Grande TC, Wilson MVH (2016). Fishes of the world.

[CR110] Nolf D (1988). Fossiles de Belgique. Dent de requins et de raies du Tertiaire de la Belgique.

[CR111] Noubhani A, Cappetta H (1997). Les Orectolobiformes, Carcharhiniformes et Myliobatiformes (Elasmobranchii, Neoselachii) des bassins phosphate du Maroc (Maastrichtien-Lutetien basal). Systematique, biostratigraphie, evolution et dynamique des faunes. Palaeo Ichthyologica.

[CR112] Otero RA, Oyarzún JL, Soto-Acuña S, Yury-Yáñez RE, Gutierrez NM, Le Roux JP, Torres T, Hervé F (2013). Neoselachians and Chimaeriformes (Chondrichthyes) from the latest Cretaceous-Paleogene of Sierra Baguales, southernmost Chile. Chronostratigraphic, paleobiogeographic and paleoenvironmental implications. Journal of South American Earth Sciences.

[CR113] Otero RA, Soto-Acuña S (2015). New chondrichthyans from Bartonian-Priabonian levels of Río de Las Minas and Sierra Dorotea, Magallanes Basin, Chilean Patagonia. Andean Geology.

[CR114] Pagani M, Zachos JC, Freeman KH, Tipple B, Bohaty S (2005). Marked decline in atmospheric carbon dioxide concentrations during the Paleogene. Science.

[CR115] Papazzoni CA, Carnevale G, Fornaciari E, Giusberti L, Trevisani E, Papazzoni CA, Giusberti L, Carnevale G, Roghi G, Bassi D, Zorzin R (2014). The Pesciara-Monte Postale Fossil-Lagerstätte: 1. Biostratigraphy, sedimentology and depositional model. The Bolca Fossil-Lagerstätte: A window into the Eocene World.

[CR116] Papazzoni CA, Fornaciari E, Giusberti L, Vescogni A, Fornaciari B (2017). Integrating shallow benthic and calcareous nannofossil zones: the Lower Eocene of the Monte Postale section (northern Italy). Palaios.

[CR117] Papazzoni CA, Giusberti L, Trevisani E, Papazzoni CA, Giusberti L, Carnevale G, Roghi G, Bassi D, Zorzin R (2014). The Pesciara-Monte Postale Fossil-Lagerstätte: 10. The Spilecco site. The Bolca Fossil-Lagerstätte: A window into the Eocene World.

[CR118] Papazzoni CA, Trevisani E (2006). Facies analysis, palaeoenvironmental reconstruction, and biostratigraphy of the “Pesciara di Bolca” (Verona, northern Italy): An early Eocene Fossil- Lagerstätte. Palaeogeography, Palaeoclimatolology, Palaeoecology.

[CR119] Parmley D, Cicimurri DJ (2005). First record of a chimaeroid fish from the Eocene of the southeastern United States. Journal of Paleontology.

[CR120] Patterson C (1993). An overview of the early fossil record of acanthomorphs. Bulletin of Marine Science.

[CR121] Pearson PN, McMillan IK, Wade BS, Jones TD, Coxall HK, Bown PR, Lear CH (2008). Extinction and environmental change across the Eocene-Oligocene boundary in Tanzania. Geology.

[CR122] Pfaff C, Zorzin R, Kriwet J (2016). Evolution of the locomotory system in eels (Teleostei: Elopomorpha). BMC Evolutionary Biology.

[CR123] Pimiento P, Ehret DJ, MacFadden BJ, Hubbell G (2010). Ancient nursery area for the extinct giant shark Megalodon from the Miocene of Panama. PLoS One.

[CR124] Popov EV, Lapkin AV (2000). A new shark species of the genus *Galeorhinus* (Chondrichtyes, Triakidae) from the Cenomanian of the lower Volga River Basin. Paleontologičeskii žurnal.

[CR125] Popov YV, Yarkov AA (2001). A new giant species of *Edaphodon* (Holocephali: Edaphodontidae) from the Beryozovaya Beds (Lower Paleocene) of the Volgograd Volga Region. Paleontological Journal.

[CR126] Prothero DR, Ivany LC, Nesbitt E (2003). From greenhouse to Icehouse: The marine Eocene-Oligocene transition.

[CR158] Rafinesque, C.S. 1810. *Caratteri di alcuni nuovi generi e nuove specie di animali e pinate della Sicilia, con varie osservazioni sopra i medisimi, lère partie*. Palermo: Per le stampe di S. Filippo.

[CR127] Rana RS, Kumar K, Singh H (2004). Vertebrate fauna from the subsurface Cambay Shale (Lower Eocene), Vastan Lignite Mine, Gujarat, India. Current Science.

[CR128] Rayner R, Mitchell T, Rayner M, Clouter F (2009). London clay fossils of Kent and Essex.

[CR129] Rosenberger LJ (2001). Phylogenetic relationships within the stingray genus *Dasyatis* (Chondrichthyes: Dasyatidae). Copeia.

[CR130] Sallan L, Coates M (2014). The long-rostrumed elasmobranch *Bandringa* Zangerl, 1969 and taphonomy within a Carboniferous shark nursery. Journal of Vertebrate Paleontology.

[CR131] Schauroth CF (1865). Verzeichnis der Versteinerungen im Herzoglichen Naturaliencabinet zu Coburg.

[CR132] Sorbini L (1972). I Fossili di Bolca.

[CR133] Stahl B (1999). Handbook of Paleoichthyology 4—Chondrichthyes III: Holocephali.

[CR135] Takeuchi GT, Huddleston RW (2006). A Miocene chimaeroid fin spine from Kern County, California. Bulletin of the Southern California Academy of Sciences.

[CR136] Tyler JC, Santini F (2002). Review and reconstructions of the tetraodontiform fishes from the Eocene of Monte Bolca, Italy, with comments on related Tertiary taxa. Studi e Ricerche sui Giacimenti Terziari di Bolca.

[CR137] Underwood CJ, Ward DJ, King C, Antar SM, Zalmout IS, Gingerich PD (2011). Shark and ray faunas in the Middle and Late Eocene of the Fayum Area, Egypt. Proceedings of the Geologists’ Association.

[CR138] Vescogni A, Bosellini FR, Papazzoni CA, Giusberti L, Roghi G, Fornaciari E, Dominici S, Zorzin R (2016). Coralgal buildups associated with the Bolca Fossil-Lagerstätten: new evidence from the Ypresian of Monte Postale (NE Italy). Facies.

[CR139] Volta GS (1796). Ittiolitologia Veronese del Museo Bozziano ora annesso a quello del Conte Giovambattista Gazola e di altri gabinetti di fossili veronesi.

[CR162] Ward, D.J. 1973. The English Palaeogene chimaeroid fishes. *Proceedings of the Geologists' Association* 84 (3): 315–330.

[CR141] Ward DJ, Grande L (1991). Chimaeroid fish remains from Seymour Island. Antarctic Peninsula. Antarctic Science.

[CR156] Winkler, T.C. 1874. Mémoire sur des dents de poisons du terrain bruxellien. *Archives du Musée Teyler *3: 295–304.

[CR143] Woodward AS (1889). Catalogue of the fossil fishes in the British Museum. Part. I. Elasmobranchii.

[CR144] Woodward AS (1899). Notes on the teeth of sharks and skates from English Eocene formations. Proceedings of the Geological Association of London.

[CR154] Woodward, A.S., and E.I. White. 1930. On Some New Chimaeroid Fishes from Tertiary Formations. *Annals and Magazine of Natural History* 6(35): 577–582.

[CR145] Zachos JC, Dickens GR, Zeebe RE (2008). An early Cenozoic perspective on greenhouse warming and carbon-cycle dynamics. Nature.

[CR146] Zachos JC, Pagani M, Sloan L, Thomas E, Billups K (2001). Trends, rhythms, and aberrations in global climate 65 Ma to present. Science.

[CR147] de Zigno A (1874). Catalogo Ragionato dei Pesci Fossili del Calcare Eoceno di M. Bolca e M. Postale.

[CR148] de Zigno A (1874). Annotazioni paleontologiche. Pesci fossili nuovi del calcare eoceno dei monti Bolca e Postale. Memorie del Reale Istituto Veneto di Scienze, Lettere ed Arti.

[CR149] de Zigno A (1876). Annotazioni paleontologiche. Aggiunte alla ittiologia dell’epoca eocena. Memorie del Reale Istituto Veneto di Science, Lettere ed Arti.

[CR150] de Zigno A (1885). Sopra uno scheletro fossile di *Myliobatis* esistente nel museo Gazola in Verona. Memorie del Reale Istituto Veneto di Scienze, Lettere ed Arti.

